# Fundamental Aspects and Comprehensive Review on Physical Properties of Chemically Grown Tin-Based Binary Sulfides

**DOI:** 10.3390/nano11081955

**Published:** 2021-07-29

**Authors:** Sreedevi Gedi, Vasudeva Reddy Minnam Reddy, Tulasi Ramakrishna Reddy Kotte, Chinho Park, Woo Kyoung Kim

**Affiliations:** 1 School of Chemical Engineering, Yeungnam University, 280 Daehak-ro, Gyeongsan 38541, Korea; drsrvi9@gmail.com (S.G.); chpark@ynu.ac.kr (C.P.); 2 Department of Physics, Sri Venkateswasra University, Tirupati 517 502, India; ktrkreddy@gmail.com

**Keywords:** o-SnS, c-SnS, SnS_2_, Sn_2_S_3_, CBD, solar cells

## Abstract

The rapid research progress in tin-based binary sulfides (Sn_x_S_y_ = o-SnS, c-SnS, SnS_2_, and Sn_2_S_3_) by the solution process has opened a new path not only for photovoltaics to generate clean energy at ultra-low costs but also for photocatalytic and thermoelectric applications. Fascinated by their prosperous developments, a fundamental understanding of the Sn_x_S_y_ thin film growth with respect to the deposition parameters is necessary to enhance the film quality and device performance. Therefore, the present review article initially delivers all-inclusive information such as structural characteristics, optical characteristics, and electrical characteristics of Sn_x_S_y_. Next, an overview of the chemical bath deposition of Sn_x_S_y_ thin films and the influence of each deposition parameter on the growth and physical properties of Sn_x_S_y_ are interestingly outlined.

## 1. Introduction

**Figure 1:**
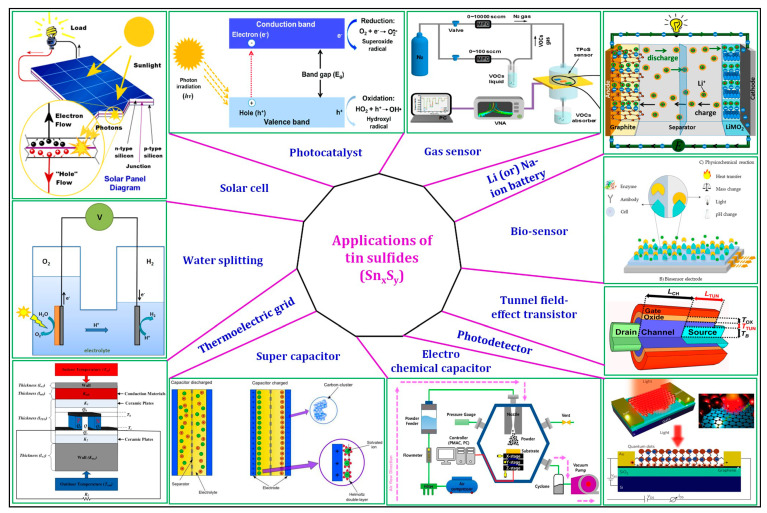
Applications of Sn_x_S_y_ [4,5,6,7,8,9,10,11,12]. Solar cell (o-SnS, c-SnS, Sn_2_S_3_ as light absorber and SnS_2_ as buffer); photodetector (o-SnS, c-SnS, Sn_2_S_3_ as light absorber and SnS_2_ as buffer); Li- and Na-ion batteries (o-SnS, c-SnS, and SnS_2_ as anode materials); gas- and bio sensors (o-SnS, c-SnS, and SnS_2_ as sensing materials); tunnel field-effect transistors (TFET) (o-SnS, c-SnS, and SnS_2_ as top or back gates); electrochemical and super capacitors (o-SnS, c-SnS, and SnS_2_ as electrode materials); capacitor; thermoelectrics (o-SnS, c-SnS, and Sn_2_S_3_ as grids); and water-splitting (o-SnS, c-SnS, and SnS_2_ as photocathodes).

To make a significant contribution to the energy needs of society with low production cost, thin film photovoltaic (TFPV) technology has been developed. Currently, the CdTe/CdS and Cu(In,Ga)Se_2_(CIGS)/CdS heterojunction TFPV technologies have received worldwide attention because these solar cells achieved record efficiencies of 22.1% [1] and 23.35% [2], respectively. However, their wider impact is hindered due to major concerns raised on the presence of harmful elements (Cd and Se) and scarcity of constituent elements (Te, In and Ga). In view of that, considerable efforts have been made to develop environmentally friendly absorbers and buffers that are free from the aforementioned toxic and inadequacy elements. Along this path, the tin-based binary sulfides (Sn_x_S_y_) such as tin monosulfide (orthorhombic (ORT)-SnS and cubic (CUB)-SnS), tin disulfide (SnS_2_), and tin sesquisulfide (Sn_2_S_3_) have drawn much attention because these are abundant, inexpensive, and nontoxic [3]. According to the merits of Sn_x_S_y_ (see Table 1), the o-SnS, c-SnS, and Sn_2_S_3_ are strongly expected as potential and alternative absorbers to the conventional CdTe and CIGS, and SnS_2_ is expected as an appropriate and alternative buffer to the regular CdS. Furthermore, their simple composition and promising physical properties made them suitable for other applications such as photocatalytic, thermoelectric, etc. (see Figure 1). Among Sn_x_S_y_, o-SnS, SnS_2_, and Sn_2_S_3_ occur naturally, whereas c-SnS was synthesized in the laboratory. The historical information and the applications of Sn_x_S_y_ available in the literature are presented in Table 2.

The deposition of Sn_x_S_y_ in thin film form became prominent owing to their wide applications. The selection of deposition technique along with the growth conditions is critical because the properties of Sn_x_S_y_ thin films are susceptible to their growth method. Sn_x_S_y_ films should be prepared by low-cost techniques such as solution processes to further reduce the production cost of TFPV devices. The preparation of Sn_x_S_y_ thin films via chemical methods, especially by chemical bath deposition(CBD), includes a slightly low cost and is, in fact, unchallenging on the preparation side. In addition, CBD provides several experimental flexibilities such as non-vacuum thin-film deposition, a wide selection of various substrates, easy doping of elements, and room temperature film growth, which are suitable for the large-scale fabrication of flexible devices for industrial applications.

The deposition of Sn_x_S_y_ thin films using CBD is relatively new, and their process–property relationships must be understood for the desired application. Further, the formation of single-phase o-SnS, c-SnS, SnS_2_, and Sn_2_S_3_ thin films in CBD is highly dependent on preparative conditions. In addition, identification and separation of the o-SnS, c-SnS, SnS_2_, and Sn_2_S_3_ phases are also critical criteria. However, there is a lack of comprehensive studies on the optimization of growth parameters until now. Therefore, extensive research studies on the growth, deposition mechanism, and preparative parameters that affect phase separation and the physical properties of Sn_x_S_y_ thin films are crucial to a successful device design in the production of clean energy.

In this scenario, the present article provides an overview of the bulk properties of Sn_x_S_y_ and a comprehensive review of the deposition, growth mechanism, and effect of growth parameters on the physical properties of Sn_x_S_y_ thin films. According to the authors’ knowledge, this is the first review of Sn_x_S_y_ thin films by CBD.

## 2. Physical Properties of o-SnS, c-SnS, SnS_2_, and Sn_2_S_3_

The physical properties such as structural, optical, and electrical properties of Sn_x_S_y_ can significantly influence the device’s performance. The crystal structure of a material can influence its optical and electrical properties, which can affect the material-related device performance [92]. Understanding and obtaining knowledge on the electronic band structure and optical characteristics of Sn_x_S_y_ is essential before using them for device applications because the main optical parameter, band gap energy (E_g_), is very sensitive to the crystal structure and defects, which directly influences the performance of a PV device. Electrical characteristics such as conduction type, resistivity, carrier concentration, and mobility of Sn_x_S_y_ play a key role in achieving high-performance photovoltaic devices. These electrical properties critically depend on the formation of defects in Sn_x_S_y_. Therefore, a good understanding of the physical properties is required for the development of effective devices.

### 2.1. Crystal Structure and Structural Characteristics

**Figure 2:**
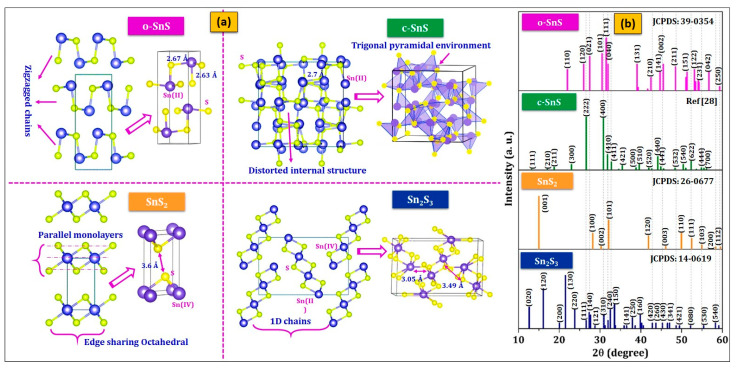
(**a**). Crystal structures of ground state Sn_x_S_y_ forms (reprinted with permission [51] © 2017, Royal Society of Chemistry) and (**b**) standard powder diffraction patterns for Sn_x_S_y_.

In o-SnS, c-SnS, SnS_2_, and Sn_2_S_3_, Sn exhibits ‘2+’ (divalent Sn(II)) or/and ‘4+’ (tetravalent Sn(IV)) oxidation states (Table 3). The capability of Sn to elect different oxidation states is the origin of structural diversity/different structures (Figure 2a) of resulting compounds [93]. o-SnS (α-SnS, space group, Pnma-$D_{16}^{2h}$) and c-SnS (π-SnS, space group, P2_1_3) are the two polymorphic forms of SnS, resulting from distortions in the crystal lattice depending on growth conditions. The o-SnS consists of a double layer of Sn and S atoms (two-dimensional SnS sheets) with zigzagged chains in which each Sn atom bonds to two S atoms in the b–c plane of the layer with a bond length of 2.671 Å and one additional S atom at a short bond length of 2.633 Å perpendicular to the plane (along a-axis) in the layer stack. The interlayer bond length of Sn-Sn atoms is 3.48 Å, and the distance between the layer stacks is 2.79 Å. The lone pair of 5s^2^ in the Sn atom occupies the fourth coordination site and weakens interaction along the b-axis. In the case of c-SnS, each Sn atom bonds with three nearest S atoms at 2.7 Å and forms a trigonal pyramidal environment, with the Sn-S bond at the trigonal base and the 5s^2^ lone pair pointing toward the apex. The stereo chemical activity of Sn(II) 5s^2^ lone pair creates a highly distorted internal structure of c-SnS. The local coordination in c-SnS is similar to o-SnS; however, a three-dimensional network is formed by a covalent bond [60]. Additionally, SnS also exhibits some of other polymorphs [94] such as, β-SnS (formed at T > 880 K), γ-SnS [95], δ-SnS [96,97], RS-SnS (rock salt-SnS, Fm$\bar{3}$m) [98]. One key point is that c-SnS and RS-SnS belong to the cubic crystal system. Further, c-SnS is a simple cubic lattice type, and RS-SnS is a face-centered cubic lattice (important note: the structure of c-SnS was incorrectly assigned as ZB-SnS previously in the literature. To avoid confusion in the literature, the readers should replace ZB-SnS with c-SnS in previous literature). More details can be found in the literature clarifying these assignments [28,93,99,100]. Furthermore, the weak interactions between distorted lone pair of 5s^2^ in Sn and neighboring S form the metastable SnS crystals or polymorphs of SnS [101].

Next, the SnS_2_ adopts a layered hexagonal structure with P$\bar{3}$ml space group, similar to the structure of the CdI_2_ system. In this structure, the layers are arranged in the b–c plane, which is perpendicular to the a-axis, and each layer is composed of S, Sn, and S atomic parallel monolayers. Each Sn atom forms bonds in an octahedral environment with six S atoms, similar in rutile SnO_2_ structure [93]. It has a symmetric edge-sharing Sn(IV)S6 octahedral with 2D planes that are separated by weak van der Waals interaction between 3.6 Å distant S atoms [61]. Finally, the Sn_2_S_3_ exhibits an orthorhombic crystal structure similar to o-SnS with the same space group of Pnma. It contains tetravalent (4+) and divalent (2+) Sn atoms in equal proportions, and they form Sn(IV)S_6_ octahedral 1D chains covered by Sn(II) tetrahedral [51]. The lone pair in Sn(II) occupies one coordination site as in the ground state form of o-SnS. These lone pairs are responsible for weak interchain interactions. The optimized theoretical lattice parameters along with experimental values for all these Sn_x_S_y_ phases are presented in Table 3. The structural characterization of Sn_x_S_y_ thin films is generally performed by X-ray diffraction (XRD). The standard XRD patterns of o-SnS, c-SnS, SnS_2_, and Sn_2_S_3_ (Figure 2b) showed the highest intensity for peaks located at 2θ of: 31.53°, 26.63°/30.84°, 15.03°, and 21.49°, arising from diffraction from (111), (222)/(400), (001), and (130) planes, respectively [25,28,102,103,104].

**Figure 3:**
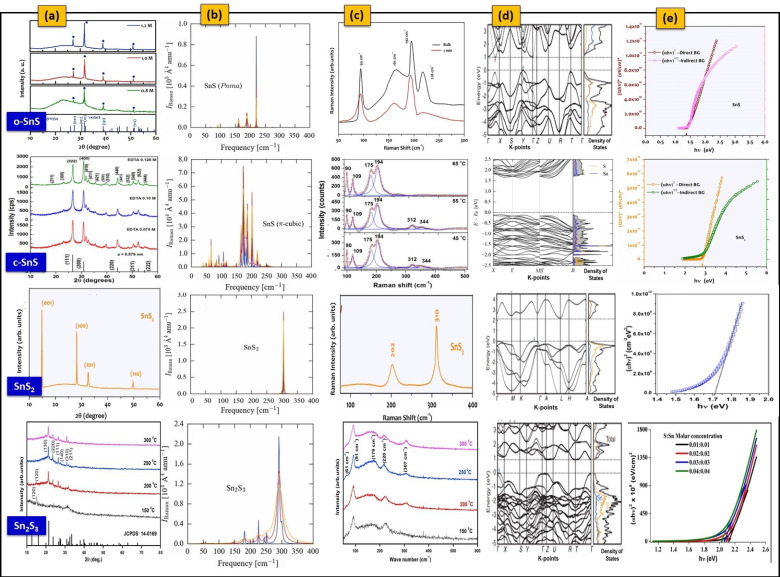
(**a**) XRD profiles of o-SnS and c-SnS (reprinted with permission [26]. © 2016, Elsevier), hexagonal SnS_2_ (reprinted with permission [123]. © 2016, Elsevier), and orthorhombic Sn_2_S_3_ (reprinted with permission [113]. © 2016, Elsevier). (**b**) Simulated Raman spectra for Sn_x_S_y_ at different temperatures of 10 K, 150 K, and 300 K (reprinted with permission [51]. © 2017, Royal Society of Chemistry). (**c**) Experimental Raman spectra of o-SnS (reprinted with permission [110]. © 2017, Elsevier), c-SnS (reprinted with permission [26]. © 2016, Elsevier), hexagonal SnS_2_ (reprinted with permission [123]. © 2016, Elsevier), and orthorhombic Sn_2_S_3_ (reprinted with permission [113]. © 2016, Elsevier). (**d**) Band structures of Sn_x_S_y_ (reprinted with permission [114]. © 2016, American Physical Society), and (**e**) bandgap estimation of o-SnS (reprinted with permission [123]. © 2016, Elsevier), c-SnS (reprinted with permission [26]. © 2016, Elsevier), hexagonal SnS_2_ (reprinted with permission [123]. © 2016, Elsevier), and orthorhombic Sn_2_S_3_ (reprinted with permission [124]. © 2016, Sciendo).

The o-SnS, c-SnS, SnS_2_, and Sn_2_S_3_ thin films have similarities in XRD patterns (Figure 3a). Thus, clear differentiation of one phase to another is difficult by using diffraction analysis alone. In this respect, it is preferable to identify these phases in pure form using a complementary method such as Raman spectroscopy because the Raman spectrum is sensitive to mainly crystal quality, structural symmetry, and strength of the chemical bond between atoms [105]. The o-SnS has 21 optical vibrational modes with the irreducible representation of Γ = 4A_g_ + 2B_1g_ + 4B_2g_ + 2B_3g_ + 2A_u_ + 4B_1u_ + 2B_2u_ + 4B_3u_ [106,107]. In these phonons, two are inactive (2A_u_), seven are infrared-active (3B_1u_, 3B_3u_, and 1B_2u_), and twelve are Raman-active (4A_g_, 2B_1g_, 4B_2g_, and 2B_3g_). In the case of c-SnS, there are 189 optic branches, and they can be reduced to 3 in the form of Γ = 16A + 16E + 47T [51]. Next, the SnS_2_ has six vibrational modes with the irreducible representation, Γ = A_1g_ + E_g_ + 2A_2u_ + 2 E_u_ [108]. The six optic modes are divided into two Raman-active modes (A_1g_ and E_g_), two infrared-active (A_2u_ and E_u_), and two acoustic modes (A_2u_ and E_u_). Additionally, the Sn_2_S_3_ has 57 optic modes with reduced form of Γ = 10A_g_ + 5A_u_ + 5B_1g_ + 9B_1u_ + 10B_2g_ + 4B_2u_ + 5B_3g_ + 9B_3u_ [51,109]. The simulated Raman spectra [51] (Figure 3b) clearly showed the significant differences in frequencies and spectral intensities because the o-SnS, c-SnS, SnS_2_, and Sn_2_S_3_ phases have differences in structure and bonding. The Raman spectrum of the o-SnS showed three prominent peaks at 160 cm^−1^ (narrow mode, B_2g_), 189 cm^−1^ (highest intensity mode, A_g_), and 220 cm^−1^ (narrow mode, A_g_). The spectrum also showed a weak A_g_ mode at approximately 92 cm^−1^, which has a narrow line width up to room temperature. In the case of c-SnS, there are three strong A phonon modes at 174 cm^−1^, 187 cm^−1^, and 202 cm^−1^ and two prominent E modes at 166 cm^−1^ and 183 cm^−1^ along with a group of weak modes in between the ranges of 50–125 cm^−1^ and 200–250 cm^−1^. Next, the SnS_2_ showed a single and strong mode at 305 cm^−1^ (A_g_), which has a constant line width with the temperature. Furthermore, the Sn_2_S_3_ showed a significantly high-intensity A_g_ mode at 291 cm^−1^ and a moderate-intensity A_g_ mode at 300 cm^−1^ with a narrow line width. Its spectrum also showed weak modes at 182 cm^−1^, 210 cm^−1^, 226 cm^−1^, 244 cm^−1^, and 252 cm^−1^, which are observed at high temperatures. Notably, the Raman mode of Sn_2_S_3_ (307 cm^−1^) overlaps marginally with the active mode of SnS_2_ (310 cm^−1^). However, the phase can be easily identified based on the band width of modes. Sn_2_S_3_ has a band width that is significantly greater than that of SnS_2_ (Figure 3b). From the experimental Raman spectra (Figure 3c), o-SnS, c-SnS, SnS_2,_ and Sn_2_S_3_ films showed Raman active modes at 93 cm^−1^, 161 cm^−1^, 192 cm^−1^, and 218 cm^−1^ [110]; 59 cm^−1^, 71 cm^−1^, 90 cm^−1^, 112 cm^−1^, 123 cm^−1^, 176 cm^−1^, 192 cm^−1^, 202 cm^−1^, and 202 cm^−1^ [111]; 224 cm^−1^ and 310 cm^−1^ [112]; and 61 cm^−1^, 91 cm^−1^, 179 cm^−1^, 220 cm^−1^, and 307 cm^−1^ [113], respectively, which matched well with theoretically calculated data. The Raman results suggested that o-SnS, c-SnS, SnS_2_, and Sn_2_S_3_ phases have distinct modes. Moreover, S-rich impurity phases can easily be found in SnS due to the sharp Raman mode of SnS_2_ (310 cm^−1^).

### 2.2. Electronic Band Structure and Optical Characteristics

According to the electronic band structures of Sn_x_S_y_ (Figure 3d), the valence band maxima (VBM) of o-SnS, c-SnS, and Sn_2_S_3_ are formed mostly of S 3p and Sn 5s hybrid states with a tiny contribution from Sn 5p states, whereas SnS_2_ is primarily composed of S 3p orbitals. The conduction band minimum (CBM) of SnS_2_ and Sn_2_S_3_ is formed by the Sn 5s bands, whereas SnS is mainly composed of Sn 5p orbitals. The VBM of SnS_2_ is lower than those of o-SnS, c-SnS, and Sn_2_S_3_; however, the CBMs of all o-SnS, c-SnS, SnS_2_, and Sn_2_S_3_ are almost aligned. The partial hybridizations with S 3s and Sn 5p states result in SnS polymorphs due to the change in density of state.

On the other hand, the band-edge positions deviated from the special points in the reciprocal space except for the CBM of Sn_2_S_3_. However, they fall between the Brillouin zone center and the zone boundaries [64,114]. From the ab initio band-structure calculations and Kohn–Sham density-functional theory, o-SnS and c-SnS exhibited the indirect and direct energy gaps of 1.6 eV and 1.8 eV; and 1.72 eV and 1.74 eV, respectively, which is due to an inherent error in calculating band structure [58]. The energy difference between direct and indirect band gaps is small; thus, the change in the nature of the band gap could be due to the effect of temperature through thermal expansion and electron-phonon coupling [64]. According to the band-structure calculations from the Hartree−Fock exchange HSE06 functional technique, o-SnS, SnS_2,_ and Sn_2_S_3_ showed indirect energy gaps of 1.11 eV, 2.24 eV, and 1.09 eV, respectively [115]. The optical characterization of Sn_x_S_y_ thin films is generally studied by a UV-Vis-NIR spectrometer. Although theoretical calculations showed the indirect band gap energy of Sn_x_S_y_, most of the experimental studies (Figure 3e) proved that Sn_x_S_y_ thin films have direct band gap energies, with the following ranges (Table 4 and Table 5): 1.16–1.79 eV; 1.64–1.75 eV; 2.04–3.30 eV; 0.95–2.03 eV; for o-SnS, c-SnS, SnS_2_, and Sn_2_S_3_, respectively. The band gap energies of these phases depend on various factors such as strain, sulfur impurities, and Sn vacancies [116,117,118,119,120,121,122].

### 2.3. Conduction Type and Electrical Characteristics

**Figure 4:**
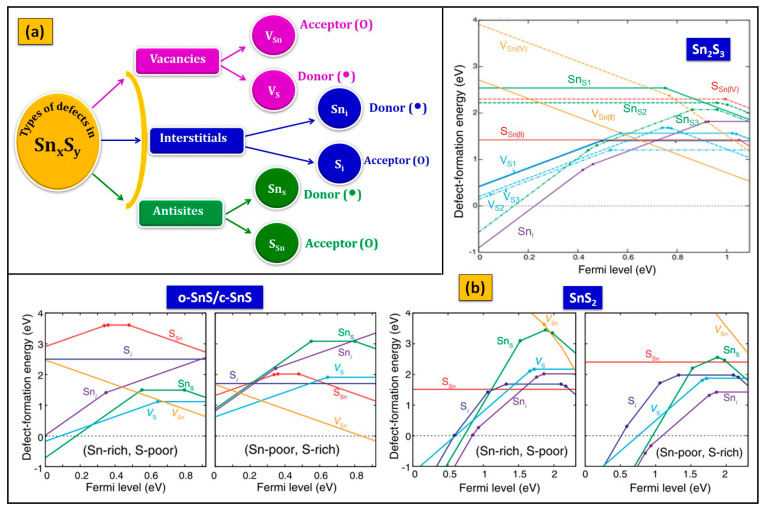
(**a**) Possibility of different defect formation in Sn_x_S_y_, and (**b**) defect formation energies in Sn_x_S_y_ as a function of Fermi energy under Sn-rich (S-poor) and Sn-poor (S-rich) conditions (reprinted with permission [114]. © 2016, American Physical Society).

In Sn_x_S_y_, three types of defects, namely, (i) Sn and S vacancies (V_Sn_ and V_S_), (ii) Sn and S interstitials (Sn_i_ and S_i_), and (iii) Sn on S antisites (Sn_S_) and S on Sn antisites (S_Sn_) are commonly formed, as shown in Figure 4a. According to the defect energy concepts (Figure 4b), the formation energy of vacancies (V_Sn(II) or Sn(IV)_, Vs) depends on the coordination number, i.e., it generally increases with increasing coordination number. Thus, V_Sn(II)_ has lower formation energy compared to V_Sn(IV)_ because the coordination number is three for Sn(II) and six for Sn(IV). As a result, V_Sn(II)_ becomes a major defect that acts as an acceptor and contributes to the p-type conducting nature to SnS. In SnS, the primary defects are V_Sn_ and V_S_, whereas Sn_i_ and S_i_ have higher energies. SnS exhibits the p-type at the Sn-poor condition, whereas the n-type at the Sn-rich condition. The defect-formation energies in SnS_2_ differ from those in SnS. The major defects are V_S_, S_i_, and S_Sn(II)_, which are inert to carrier generation. The defect-formation energies in Sn_2_S_3_ are typically interpreted as a mixture of those in SnS and SnS_2_. On the other hand, the formation energy of interstitials (Sn_i_, S_i_) associates with the gap of interlayer free spaces, and that gap follows the notation of SnS > Sn_2_S_3_ > SnS_2_. Sn_i_ always prefers to locate at the center of the gaps, whereas S_i_ likes to make a covalent bond with neighboring S atoms. The tin interstitial, Sn_i_ in both SnS_2_ and Sn_2_S_3_, has lower formation energy compared to SnS, and it acts as a deep donor in SnS_2_ and Sn_2_S_3_ and contributes to an n-type conductivity. All the antisites in Sn_x_S_y_ have higher formation energies because they are correlated with chemical bonds. Therefore, these defects do not play a major role in the conduction type of Sn_x_S_y_ phases. The electrical characterization of Sn_x_S_y_ thin films is generally performed by the popular van der Pauw–Hall method.

From the reported electrical parameters of Sn_x_S_y_ films (Table 3), the o-SnS has a hole density in the order of 10^11^–10^18^ cm^−3^, hole mobility in the range of 4–500 cm^2^ V^−1^ s^−1^, and electrical resistivity in the range of 13–10^5^ Ω cm. In contrast, the c-SnS has a hole density in the order of 10^11^–10^18^ cm^−3^, hole mobility in the range of 10^−2^–78 cm^2^ V^−1^ s^−1^, and electrical resistivity in the range of 70–10^7^ Ω cm. In the case of the SnS_2,_ it has a carrier concentration of the order of 10^13^–10^17^ cm^−3^, electron mobility in the range of 15–52 cm^2^ V^−1^ s^−1^, and electrical resistivity in the range of 1.11–10^7^ Ω cm, whereas the Sn_2_S_3_ has a carrier density in the order of 10^14^–10^16^ cm^−3^ and resistivity in the range of 0.4–10^5^ Ω cm, and a very little information related to Sn_2_S_3_ carrier mobility value is available in the literature. The reported variation in electrical parameters is expected due to the differences in the growth process and chemical composition.

## 3. Influence of Deposition Parameters on Sn_x_S_y_ Thin Film Growth and Properties

In CBD, the selection of tin source precursor, sulfur source precursor, complexing agent, and their concentrations is crucial to prepare the high-quality Sn_x_S_y_ thin films using CBD. Moreover, the selection of suitable activation conditions such as solution/bath temperature, solution/bath pH (acidic or basic medium), deposition time, and stirring speed is also important [36,142] because they significantly affect the phase formation, growth, and properties of Sn_x_S_y_ films. In addition to the above parameters, the nature of the substrate and its cleaning procedure also affect the phase formation, growth, and properties of Sn_x_S_y_ films. Therefore, the understanding of the influence of all those parameters on the growth process of Sn_x_S_y_ films and their physical properties is necessary to deposit the quality films for device applications. In Table 4 and Table 5, the deposition parameters used for different thin films of tin sulfides made from chemical methods were summarized.

### 3.1. Overview of CBD Process of Sn_x_S_y_ Thin Films

**Figure 5:**
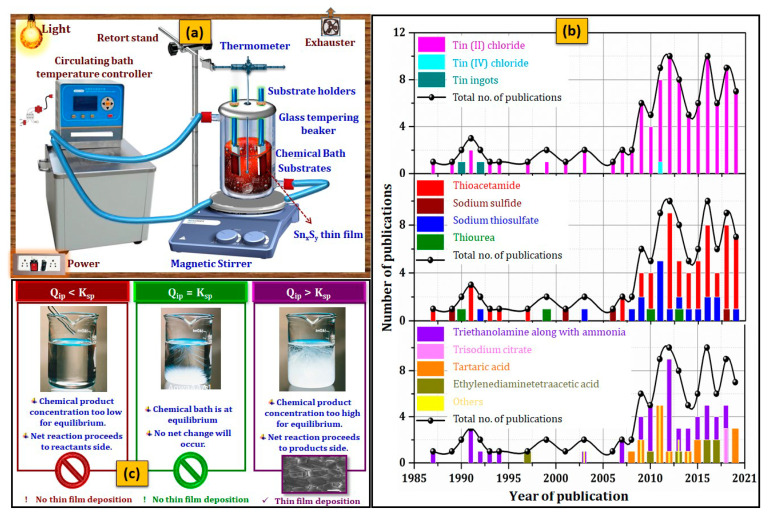
Schematic representation of (**a**) CBD, (**b**) main chemical reagents used for the preparation of Sn_x_S_y_ thin films from 1987 to the present, and (**c**) importance of K_sp_ and Q_ip_ relation on the films by CBD.

The CBD refers to “a typical synthesis employing mild conditions [143]”. As schematically illustrated in Figure 5a, the experimental setup of CBD consists of the following parts: (i) magnetic stirrer with thermostat (to stir the mixed reactant solution continuously), (ii) oil bath (to maintain the desired temperature), (iii) substrate holder (to keep the substrates stable), (iv) stock chemical solutions to compose the reaction bath (mixture of different reagent solutions and its level always remains below the outer oil level), and (v) cleaned substrates [144]. The deposition of o-SnS, c-SnS, SnS_2_, and Sn_2_S_3_ by CBD was reported in 1987 [145], 2006 [146], 1990 [36,130], and 2012 [34], respectively. Sn_x_S_y_ films were deposited using various Sn precursors such as tin (II) chloride dihydrate (SnCl_2_∙2H_2_O), tin(IV) chloride pentahydrate (SnCl_4_∙5H_2_O), and tin ingots; various S precursors such as sodium sulfide (Na_2_S), ammonium sulfide (NH_4_)_2_S, sodium thiosulfate (Na_2_S_2_O_3_), thioacetamide (C_2_H_5_NS), and thiourea (CH_4_N_2_S); and various complexing agents such as triethanolamine (C_6_H_15_NO_3_), ammonia (NH_3_)/ammonium hydroxide (NH_4_OH), ammonium fluoride (NH_4_F), ammonium citrate (C_6_H_17_N_3_O_7_), trisodium citrate (Na_3_C_6_H_5_O_7_), citric acid (C_6_H_8_O_7_), tartaric acid (C_4_H_6_O_6_), ethylenediaminetetraacetic acid (C_10_H_16_N_2_O_8_), and disodium ethylenediaminetetraacetate (C_10_H_14_N_2_Na_2_O_8_). Among the above-mentioned chemicals, tin (II) chloride, thioacetamide, and triethanolamine, along with ammonia, were widely used as Sn precursor, S precursor, and complexing agents, respectively (Figure 5b). Other types of Sn precursors such as tin ingots [130,147] and tin (IV) chloride [131] were employed to deposit the SnS_2_ films. Except for the above reports, tin (II) chloride was used as an Sn source. In the case of S precursors, sodium thiosulfate was used as a second alternative to the regularly used thioacetamide.

The preparation of Sn_x_S_y_ thin films by CBD occurs when a substrate is immersed in the solution mixture of Sn ion (Sn^2+^ or Sn^4+^)-source, S ion (S^2−^)-source, and an appropriate complexing agent. In the deposition process, the Sn^2+^/Sn^4+^ ions are complexed through the coordinated bond formation by the complexing agent, which controls the rate of reaction [148]. At super saturation condition (Ionic product, Q_ip_ > Solubility product K_sp_), Sn_x_S_y_ films can be deposited (Figure 5c). However, simply maintaining supersaturation condition in the bath will not provide acceptable quality Sn_x_S_y_ films; managing the solubility product of tin hydroxides is required because when an Sn precursor is dissolved in water, it rapidly binds with hydroxide ions, creating Sn(OH)_2_ and Sn(OH)_4_. The differences in K_sp_ values between SnS (1 × 10^−25^ mol^2^ dm^−6^), irrespective of the polymorphs, and SnS_2_ (1 × 10^−46^ mol^3^ dm^−9^) are very close to those between their hydroxides (Sn(OH)_2_ (1 × 10^−28^ mol^3^ dm^−9^) and Sn(OH)_4_ (1 × 10^−56^ mol^5^ dm^−15^)). Therefore, it is vital to monitor supersaturation with respect to an individual phase as well as the growth kinetics. In addition, the K_sp_ of Sn_x_S_y_ is affected by the concentration of precursor, solvent type, bath temperature, and bath pH [148,149]. Therefore, the optimum condition for the deposition of Sn_x_S_y_ thin film can be achieved by manipulating the above deposition parameters.

**Figure 6:**
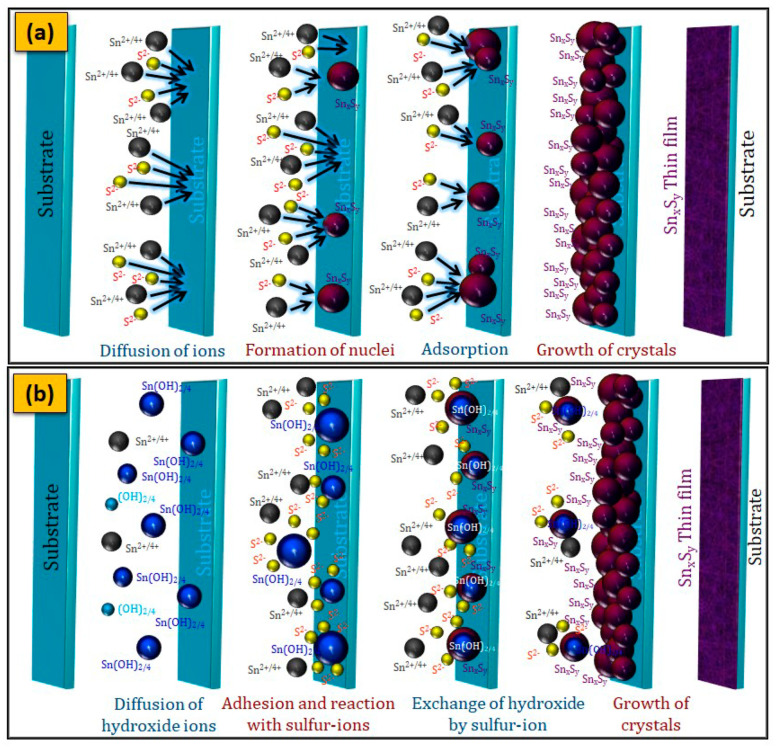
Schematic representation of (**a**) ion-by-ion mechanism and (**b**) cluster-by-cluster (hydroxide) mechanism.

According to previous reports, the formation of o-SnS, c-SnS, SnS_2_, and Sn_2_S_3_ thin films is achieved through either an ion-by-ion mechanism (Figure 6a) or a simple cluster (hydroxide) mechanism (Figure 6b) based on the reaction process and parameters maintained in the bath [150]. The formation reaction of Sn_x_S_y_ thin films through the ion-by-ion mechanism and cluster (hydroxide) mechanism is as follows:

Ion-by-ion mechanism:

xSn^p+^ + yS^q−^ → Sn_x_S_y_

[∵ p = 2 or 4, q = 2; x = 1 or 2, y = 1, 2, or 3; Sn_x_S_y_ = SnS, SnS_2_, or Sn_2_S_3_]

Cluster (hydroxide) mechanism:

xSn^p+^ + y(OH)^q−^ → Sn(OH)_n_

Sn(OH)_n_ + yS^q−^ → Sn_x_S_y_ + n(OH)

[∵ p = 2 or 4, q = 2; n = 2 or 4; x = 1 or 2, y = 1, 2, or 3; Sn_x_S_y_ = SnS, SnS_2_, or Sn_2_S_3_]

The physical properties of the CBD-deposited o-SnS, c-SnS, SnS_2_, and Sn_2_S_3_ thin films can be affected by the growth mechanism, level of supersaturation, and surface energy of the complexing agents [17]. Therefore, it is crucial to understand the actual mechanism undertaken in the solution for tuning the properties of the deposited films. Moreover, in the process of o-SnS, c-SnS, SnS_2_, and Sn_2_S_3_ thin film deposition, controlling the reaction to reduce or remove the spontaneous precipitation is essential, which can only be achieved by complexing the tin ions using an appropriate complexing agent (L). The kinetics of o-SnS, c-SnS, SnS_2_, and Sn_2_S_3_ thin film formation can be comprehended through the following reactions.

The complexing reactions in an aqueous Sn precursor solution are as follows:

SnCl_2_∙2H_2_O + L ⇔ Sn(L)^2+^ + 2Cl^−^ + 2H_2_O

SnCl_4_∙5H_2_O + L ⇔ Sn(L)^4+^ + 4Cl^−^ + 5H_2_O

The free Sn^2+^/Sn^4+^ ions are slowly released by the tin complex in a controlled way. As the tin complex dissociates, then

Sn(L)^2+^ ⇌ Sn^2+^ + L

Sn(L)^4+^ ⇌ Sn^4+^ + L

Here, the concentration of complex tin ions in the solution, Sn(L)^2+^ or Sn(L)^4+^, can be controlled by adjusting the concentration of the complexing agent and bath temperature [151]. If these ions can be generated, then the deposition of Sn_x_S_y_ thin films can be achieved. On the other hand, controlling the reaction by a slow and uniform generation of sulfur ions in the solution is also a significant factor when thin films are deposited. Thioacetamide (C_2_H_5_NS) is one of the most frequently employed S precursors. The hydrolysis of the S precursor can produce H_2_S and then S^2−^ ions by the following reactions [152]:

CH_3_CSNH_2_ + H_2_O ⇌ CH_3_CONH_2_ + H_2_S

When the reaction attains an equilibrium [153], the following reactions are expected at a temperature of 25 °C:

H_2_S + H_2_O ⇌ H_3_O^+^ + HS^−^ (K_0_ = 10^−7^)

HS^−^ ⇌ H^+^ + S^2−^ (K_1_ = 10^−17^)

HS^−^ + OH^−^ ⇌ H_2_O + S^2−^ (K_2_ = 10^−3^)

At low pH values (<2.5), the reaction is controlled by the rate of hydrolysis of S precursor leading to the formation of hydrogen sulfide (H_2_S), whereas at higher pH values (>2.5), the reaction is controlled by the formation and decomposition of the tin–thioacetamide complex. Therefore, the pH of the bath and metal–thioacetamide complexes are also considered as the growth rate- and growth mechanism-determining components in the film formation [154].

The Sn ions react with the S ions and initiate the formation of tin sulfides (o-SnS, c-SnS, SnS_2_, and Sn_2_S_3_). The generation rate of Sn and S ions is controlled primarily by the source concentration, pH, and solution temperature. When the precursor concentration is changed, multiphase or other single-phase films can be formed by the following reactions [155,156]:

Sn^2+^ + S^2−^ → SnS (K_sp_ = 1 × 10^−25^ at 25 °C)

Sn^4+^ + 2S^2−^ → SnS_2_ (K_sp_ = 1 × 10^−46^ at 25 °C)

Sn^2+^ + Sn^4+^ + 3S^2−^ → Sn_2_S_3_

Sn(II)S + Sn(I V)S_2_ → Sn(II)(IV)_2_S_3_ (or) SnS + SnS_2_ → Sn_2_S_3_

### 3.2. Sn and S Precursors and Their Concentration Effect

**Figure 7:**
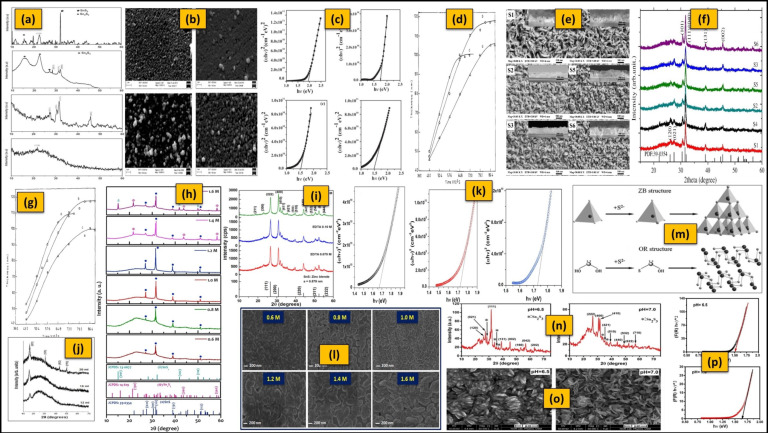
(**a**–**c**) XRD patterns, SEM images, and (αhυ)^2^ versus (hυ) graph of o-SnS films grown at various SnCl_2_ concentrations (reprinted with permission [191]. © 2012, Elsevier), and variation in o-SnS film (**d**) thickness with different TA concentrations (reprinted with permission [145]. © 1987, Elsevier). (**e**,**f**) Morphology and crystallinity changes of o-SnS films with ST concentrations (reprinted with permission [187]. © 2012, Elsevier). (**g**) Variation in SnS film thickness with different TEA concentrations (reprinted with permission [145]. © 1987, Elsevier). (**h**,**i**) XRD patterns of the o-SnS films and c-SnS films deposited at different TTA (reprinted with permission [211]. © 2019, Elsevier) and EDTA concentrations, respectively (reprinted with permission [26]. © 2016, Elsevier). (**j**) XRD patterns of SnS_2_ films deposited at various volumes of ammonia solution (reprinted with permission [41]. © 2012, Elsevier). (**k**) (αhυ)^2^ versus (hυ) for c-SnS films prepared using various EDTA amounts reprinted with permission [26]. © 2016, Elsevier). (**l**) SEM images of o-SnS films deposited with various TTA concentrations (reprinted with permission [211]. © 2019, Elsevier). (**m**,**n**) Scheme of the formation (reprinted with permission [185]. © 2011, Elsevier) and XRD patterns of o-SnS and c-SnS films (reprinted with permission [27]. © 2018, Elsevier). (**o**) Morphologies of o-SnS and c-SnS films (reprinted with permission [184]. © 2011, Elsevier), and (**p**) variation in the band gap o-SnS films at different pH values (reprinted with permission [27]. © 2018, Elsevier).

The selection of Sn precursor and its concentration plays a vital role in the growth, phase formation, crystallinity, preferred orientation, morphology, band gap, and other properties of Sn_x_S_y_ thin films [251]. This is because the releasing rate of Sn ions strongly depends on the selection of Sn precursors. As mentioned in Section 3.1, the SnCl_2_∙2H_2_O (T(II)C) has been considerably utilized as an Sn precursor for the deposition of Sn_x_S_y_ films. According to the literature (Table 4 and Table 5), until recently, there have been no reports related to the study of different types of Sn precursors on the formation of Sn_x_S_y_ films and the Sn precursor concentration effects on the formation of SnS_2_ and Sn_2_S_3_ films and their properties. However, there have been very few quantitative analyses of Sn precursor concentration effect on the formation of o-SnS films and their properties. The primary report related to the effect of Sn precursor concentration ([T(II)C] = 0.06–0.12 M) on the growth of o-SnS films was made in 2012 [191]. A lower T(II)C concentration stimulates the formation of multi phases with a dominant SnS_2_ phase, whereas a higher T(II)C concentration reduces the crystallinity. The T(II)C concentration of 0.1 M is beneficial for the deposition of pure, good crystalline o-SnS with (111) preferred orientation (Figure 7a). A small variation in T(II)C concentration (at 0.15 M) changes the preferred orientation of o-SnS from (111) to (200) [194]. Moreover, the change in T(II)C concentration can increase the grain size and decrease the band gap (1.95–1.5 eV) (Figure 7b,c) [191]. Therefore, the manipulation of preferred orientation, crystallinity, and band gap can be achieved by the change in Sn precursor concentration.

In addition to the suitable Sn precursor selection, the choice of S precursor and its concentration are highly desirable to obtain good quality Sn_x_S_y_ films. In CBD, the releasing rate (or reaction rate) of S ions greatly affect the growth kinetics and phase formation, and it can be controlled by the S precursor concentration. According to the previous reports (Table 4 and Table 5), TA and ST have been chiefly used as S ion sources (Figure 5b). In those, TA is preferable compared to ST because it works in both acidic and alkaline bath conditions. The influence of TA concentration on o-SnS film growth (thickness) was initially reported in 1987 [145]. An extremely low or high TA concentration produces the o-SnS films of smaller terminal thickness, whereas a moderate TA concentration promotes the growth of maximum thickness (Figure 7d). The reason for the lower film thickness obtained at a lower S precursor concentration is the insufficient number of S ions in the reaction bath that can combine with all the available Sn ions. At a higher S precursor concentration, the releasing rate of S ions is high enough to stimulate the precipitation process, which also results in a lower film thickness [145].

Furthermore, the S precursor concentration can influence the morphology and phase formation of o-SnS films (Figure 7e). A higher TA concentration stimulates the formation of multi phases such as Sn_2_S_3_ and Sn_3_S_4_ (Sn_2_S_3_ + SnS → Sn_3_S_4_) [209] and a lower TA concentration assists the growth of single-phase o-SnS films, but with lower crystallinity [142,198]. A TA concentration of 0.1 M is preferable for the deposition of a single-phase, polycrystalline o-SnS with (101) preferred orientation [198], and a ST concentration of 0.75 M is advisable for (111)/(040) preferred orientation (Figure 7f). The effect of changes in the S source concentration on the band gap of o-SnS films is controversial until the present. A reduction in band gap from 1.70 eV to 1.25 eV with increasing TA concentrations was reported in [200], although no significant change in band gap was found with TA concentration in [198]. On the other hand, no studies in the literature have focused on the influence of S precursor concentration on the properties of c-SnS, SnS_2,_ and Sn_2_S_3_ films.

### 3.3. Complexing Agents and Their Concentration Effect

As stated in Section 3.1, in order to develop influential Sn_x_S_y_ films, the control of the availability of Sn ions in the reaction bath is essential. It can be successfully attained by the addition of an appropriate concentration of a complexing agent [128,148]. Moreover, adhesion, morphology, crystallinity, and the deposition rate of Sn_x_S_y_ films can be significantly affected by the concentration of the complexing agent [128]. Therefore, knowledge of the behavior of complexing agents in the bath can help to obtain good quality Sn_x_S_y_ films. The behavior of complexing agents is described in terms of their stability constants (K_s_), which is the equilibrium constant for the formation of a complex in a solution [252]. It is defined for the equilibrium between an Sn ion (Sn^2+/4+^) and a ligand (L) as [148]

(1) $$K_{S}=\frac{a_{{\mathrm{Sn}}^{2+/4+}}-\;L}{a_{{\mathrm{Sn}}^{2+/4+}}+{\;a}_{L}}$$

where a is the activity of subscripted species and can be approximated by its concentration. A large value of K_s_ implies a strong binding affinity for the metal (Sn) ion, while a small value of K_s_ implies a weak binding affinity [148]. Generally, complexing agents can prevent the formation of powder/bulk precipitation of tin hydroxides in the reaction bath, and they can easily maintain the supersaturating condition. If a complexing agent has a weak binding affinity, it does not arrest the bulk precipitation of tin hydroxides. On the other hand, if it has an extremely strong binding affinity, it restricts the deposition of the desired film [253]. Therefore, in order to prevent powder/bulk precipitation of tin hydroxides, the complexing binding affinity must be intermediate.

Various complexing agents have been explored to control Sn ions depending on the bath conditions during the deposition of Sn_x_S_y_ films (Table 4 and Table 5). However, there are only a few reports on the study of complexing agent concentration. Initially, the influence of TEA complexing agent concentration on the thickness of o-SnS films was made in 1987 [145]. An optimized TEA complexing agent concentration controls the formation of o-SnS films, yielding a thick o-SnS film (Figure 7g). In addition to the growth (thickness), the change in TEA complexing agent concentration can also influence the phase formation and crystallinity of o-SnS, c-SnS, and SnS_2_ films. The lower tartaric acid (TTA) complexing agent concentration creates the weak tin complexation, leading to partial homogeneous precipitation, resulting in low-crystalline o-SnS films. As the complexing agent concentration increases, the improved tin complexation controls the reaction, yielding the formation of better crystalline films o-SnS. Over the limit, the availability of free Sn ions is reduced due to strong complexation, resulting in the formation of sulfur-rich tin phases such as Sn_2_S_3_ and SnS_2_ (Figure 7h) [211]. Single-phase, polycrystalline o-SnS films with (111) preferred orientation are produced at 1.85 M of TEA [191] and 1.4 M of TTA [211], while a c-SnS (222)/(400) is formed at 0.125 M of EDTA [26] concentrations (Figure 7i). The crystallinity of o-SnS films can be improved by replacing the lower stability (Sn^2+^-TEA) complexing agent with the higher stability (Sn^2+^-EDTA) one [198,254], due to the fact that EDTA (hexaligand) may generate a ligand more quickly than TEA (triligand) [255]. An increase in citric acid and ammonia concentration also improves the crystallinity in the case of SnS_2_ films (Figure 7j) [41,131]. The concentration of the complexing agent similarly influences the morphological and optical properties of the o-SnS, c-SnS, and SnS_2_ films. The direct optical energy gap for o-SnS films reduces with increasing complexing agent concentration (TSC, 0.06–0.08 M; TEA, 12.5–13 M; TTA, 0.6–1.4 M) from 2.16 eV to 1.17 eV [50,182,214], but rises from 1.67 eV to 1.73 eV [26] for c-SnS films with EDTA (0.075–0.125 M) (Figure 7l). The change in complexing agent concentration (TSC, TTA) improves the compactness and morphology of o-SnS films (Figure 7m). This may improve their electrical properties, such as electrical mobility (~228 cm^2^V^−1^s^−1^) and carrier concentration (~4.1 × 10^15^ cm^−3^). No previous study has examined the effect of complexing agents on the formation and physical properties of Sn_2_S_3_ films.

### 3.4. Solution pH Effect

In CBD, solution pH/bath pH (a measure of the acidity or basicity of a solution) is an important parameter because it directly affects the growth mechanism as well as reaction rate. Therefore, it can influence the formation of phases and physical properties of films [27]. In addition, the bath pH must be at a specific optimum value to maintain supersaturation condition (Q_ip_ > K_sp_, Figure 5c) for the formation of Sn_x_S_y_ films. The preparation of Sn_x_S_y_ films was reported both in acidic (pH < 7) and alkaline (pH > 7) baths (see Table 4 and Table 5). When the bath pH is varied between 1 and 14, the concentration of OH- ions increases, which results in a reduction in the concentration of free Sn^2+^ or Sn^4+^ ions in the solution. Thus, the hydroxide mechanism can predominate during film development, resulting in the creation of Sn(OH)_2 or 4_ in addition to Sn_x_S_y_. A higher bath pH, on the other hand, encourages the hydrolysis of a sulfur source precursor.

A few researchers have investigated the bath pH effect on o-SnS and c-SnS films growth and their physical properties (Table 4). The bath pH effect on the adhesion and growth rate of o-SnS films was first reported in 1989 [36]. According to this report, the good adhesion of o-SnS films on glass can be obtained with a bath pH >3. The growth rate is low at pH~7 and high at pH~10 for o-SnS films due to the formation of Sn(OH)_2 or 4_ precipitate from the hydrolysis of an Sn precursor because a part of Sn(OH)_2 or 4_ precipitate turns into Na_2_SnO_2_, which dissolves back in the solution. The change in growth rate by bath pH leads to the variation in grain size of o-SnS films [183]. The bath pH can also influence the growth mechanism, which leads to phase transformation [184,185]. An o-SnS forms at a lower pH of 6.5 via the cluster-by-cluster mechanism, whereas c-SnS forms at a higher pH of 7.0 through the ion-by-ion mechanism (Figure 7n,o) [27].

The phase transition caused by the change in bath pH leads to a change in the morphologies of the film surfaces (Figure 7o) and the energy band gaps (o-SnS: 1.51 eV and c-SnS: 1.64 eV) (Figure 7p) [27,184,185]. The increase in solution pH results in the decrease of free Sn^2+^ ion concentration as well as the concentration of OH^−^ ions, which are favorable for the hydrolysis of the S ion source [256], leading to the increase in the concentration of S^2−^ ions. Thus, the interaction of Sn^2+^ and S^2−^ ions can form the c-SnS via an ion-by-ion mechanism because the potential barrier of heterogeneous nucleation is lower than that of homogeneous nucleation [25,185]. However, these mechanisms are speculated, and direct in situ measurement evidence such as in situ quartz crystal microbalance and electrochemical impedance is lacking. Therefore, such studies are required for understanding the growth mechanism of Sn_x_S_y_ films [257]. On the other hand, no studies in the literature have examined the effect of bath pH on the properties of SnS_2_ and Sn_2_S_3_ films.

### 3.5. Solution Temperature (T_b_) Effect

In CBD, solution temperature/bath temperature (T_b_) also played a crucial role in the preparation of thin films with high quality and desired features. It critically enhances the rate of dissociation of the precursors and thus strongly affects the thickness, growth rate, type of nucleation, crystalline phase, crystallite size, morphology, and optoelectrical properties of thin films. The change in film growth rate as a function of T_b_ can be determined through the Arrhenius equation [258],

(2) $$k\left(T\right)={\mathrm{Ae}}^{\frac{-E_{a}}{\mathrm{RT}}}$$

where k(T) is the temperature-dependent growth rate for the given deposition conditions, A is a pre-exponential constant related to the initial reagent concentration, E_a_ is the activation energy (kJ/mol), and R is the gas constant (R = 8.3145 J mol^−1^ K^−1^).

**Figure 8:**
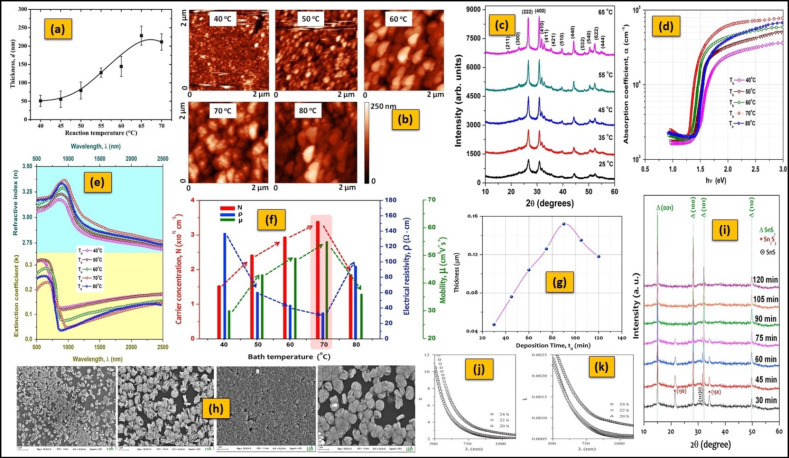
(**a**) Variation in o-SnS film thickness with bath temperature (reprinted with permission [213]. © 2019, Elsevier). (**b**,**f**) Morphological and electrical properties of o-SnS at different bath temperatures (reprinted with permission [212]. © 2019, Elsevier). (**c**) Change in crystallinity of c-SnS films with bath temperature (reprinted with permission [50]. © 2016, Elsevier). (**d**,**e**) Plots of absorption coefficient refractive index and extinction coefficient of o-SnS films (reprinted with permission [17]. © 2015, Elsevier). (**g**,**i**) Variation in thickness and crystallinity of SnS_2_ films with deposition time (reprinted with permission [56]. © 2017, Elsevier). (**h**) Morphology of ORT- SnS thin films deposited at various times (reprinted with permission [177]. © 2010, Elsevier). (**j**) Variation in refractive index and (**k**) variation in extinction coefficient of Sn_2_S_3_ films with deposition time (reprinted with permission [34]. © 2012, Elsevier).

The deposition of Sn_x_S_y_ films has been reported in the T_b_ range of room temperature (T_r_)—90 °C (Table 4 and Table 5). The effect of T_b_ on the formation of o-SnS, c-SnS films, and their properties (Table 4) was studied extensively. The T_b_ changes the growth rate due to the variation in the deposition mechanism, i.e., the ion-by-ion mechanism, which is less thermally activated with low activation energy (at lower bath temperatures). In contrast, cluster-by-cluster is believed to occur at relatively higher temperatures [259]. Thus, the thickness of a film has a close relationship with the T_b_. First, it increases significantly with the T_b_ due to the increase in bath supersaturation [145,213] and reaches saturation point very quickly because the hydrolysis of the S precursor is greatly improved by the increase in T_b_ [182] (Figure 8a). Then, it decreases down to a terminal point because of the ion–ion condensation process and high homogeneous precipitation rate [145,146]. In addition, the T_b_ significantly affects the microstructures of o-SnS and c-SnS films.

Generally, the films prepared at lower T_b_ have smaller grains, and those grains increase in size with T_b_ due to the covering of voids by secondary nucleation (Figure 8b) [50,212]. The change in grain shape may indicate a change in the growth mechanism [146]. The T_b_ can also influence the composition of c-SnS films. The c-SnS films show a non-stoichiometric composition at higher and lower Tb values due to the relatively faster and slower release of Sn^2+^ ions from the tin complex due to the variation of thermal energy in the solution [50]. Single-phase, polycrystalline o-SnS films with (111) preferred orientation and c-SnS films with (222)/(400) preferred orientation are produced separately at T_b_ of 70 °C [212] and 65 °C [50] using a different source of materials (Table 4), respectively.

On the other hand, the T_b_ shows an impact on phase transformations when other bath parameters remained constant. The films predominantly exhibit the o-SnS phase above the T_b_ range of 30–40 °C, whereas the c-SnS phase is below this range (20–30 °C) for particular deposition conditions [146]. The T_b_ can directly affect the crystallinity of both o-SnS and c-SnS films. The crystallinity of both films is improved with T_b_ due to the supply of sufficient thermal energy for further crystallization (Figure 8c) [50,212]. Thus, an average crystallite size is improved with T_b_—however, up to a certain extent [212]. As the T_b_ improves the kinetic energy of the reactants and accelerates the interaction between all ions in the reaction bath, the nuclei formation (crystallite grow) is enhanced on the surface of the substrate [260]. However, at higher T_b_, the crystallite size is decreased due to the dissolution of grown film.

The T_b_ also influences the optical characteristics of the o-SnS and c-SnS films. In o-SnS films, the T_b_ improves the sharpness of the absorption edge with a high optical absorption coefficient (>10^4^ cm^−1^) [17] (Figure 8d), which is suitable for PV devices. The optical energy gap of o-SnS and c-SnS films decreases from 1.41 eV to 1.30 eV and from 1.74 eV to 1.68 eV, respectively, with the increase in T_b_ (30–70 °C) [17,50]. As mentioned above, T_b_ can improve the grain size and simultaneously reduce height (smoothness of the surface) and the number of grain boundaries [261]; this minimizes imperfections in the film and enhances the quality of the film, which can lead to change in density of localized states within the energy gap [262]. Therefore, band gap tuning is easily possible in CBD deposited o-SnS and c-SnS films regarding T_b_, which is essential for designing highly efficient solar cells [263]. The optical parameters, namely, refractive index (n), extinction coefficient (k), and real/imaginary dielectric constants of o-SnS films, are in ranges of 2.72–3.24, 0.24–0.13, and 7.34–10.48/0.85–1.32 [17], respectively (Figure 8e). Here, the variation in the optical parameters may be arrived from the change in strain and packing density with T_b_ [264].

As previously mentioned, T_b_ improves the crystallinity along with grain size and thickness. Thus, the scattering of charge carriers by grain boundaries decreases with respect to T_b_, which makes a significant change in the electrical characteristics of films [265]. These possible reasons may improve the carrier density and a consequent reduction in resistivity in both o-SnS and c-SnS films. The reduction of the dispersing effects of carriers can lead to an increase in the mobility of carriers in those films (~55 cm^2^ V^−1^ s^−1^ for o-SnS at 70 °C [212] and 28 cm^2^ V^−1^ s^−1^ for c-SnS at 45 °C [50]) (Figure 8f). However, the above-mentioned description confirms the importance of T_b_ in the CBD process until there are no reports on the T_b_ influence on both SnS_2_ and Sn_2_S_3_ films.

### 3.6. Deposition Time Effect

Deposition time (t_d_) is the most important bath parameter among the various deposition conditions. It affects the growth rate/thickness and properties of Sn_x_S_y_ films. In CBD, the t_d_ period can be divided into three steps, namely, (i) nucleation or initiation, which requires high activation energy; (ii) linear growth, which includes heterogeneous growth of nuclei; and (iii) termination or saturation, in which chemical reagents become depleted and the reaction begins to slow down and eventually stops [266]. Thus, the rate of formation of nuclei can be in terms of t_d_ by the following Avrami equation (a conventional diffusion-controlled reaction model) [267]:

(3) $$\alpha =1-e^{-{\left({\mathrm{kt}}_{d}\right)}^{n}}$$

where α is the fractional decomposition (or reaction), k is a rate constant, and n is the Avrami exponent.

There are a considerable number of reports on the study of the t_d_ effect on o-SnS films, but there are only a few reports for c-SnS [210,225], SnS_2_ [56], and Sn_2_S_3_ [34] films (Table 4 and Table 5). Typically, a t_d_ from a few minutes to several hours has been reported to prepare these Sn_x_S_y_ films (Table 4 and Table 5). The growth of Sn_x_S_y_ films with t_d_ was simply described in terms of thickness. In the initial state, the change in film thickness is insignificant because of the requirement of long incubation time for nucleation [198], and the thickness increases linearly due to the availability of sufficient amounts of Sn^2+^ or Sn^4+^ and S^2−^ ions. Next, the film thickness increases faster, then decreases at a longer deposition time, and attains a maximum value as a terminal/final thickness. Here, the attained terminal thickness is not only t_d_-dependent but also T_b_-related [145]. Thus, a terminal thickness should be considered when the reaction undergoes at a constant temperature. A terminal thickness in the range of 120–900 nm can be obtained for different t_d_ varying from 1 h to 24 h at a constant range of T_b_ (T_r_—75 °C) for o-SnS and c-SnS films [128,145,175,198,225], and a thickness of 152 nm can be attained at a t_d_ of 90 min for SnS_2_ films (Figure 8g) [56]. The variation in the thickness (growth) of these films with respect to t_d_ can be explained by considering two competing processes taking place in the deposition bath. One process includes heterogeneous precipitation, which leads to film growth (thickness improves). The other involves the dissolution of the preformed film, which results in the decrease of film thickness.

The t_d_ has a significant impact on the surface morphology, crystallinity, crystallite size, and phase purity of Sn_x_S_y_ films. As the t_d_ increases, the size and quantity of grains (or aggregations) can be improved to form a more homogeneous film [177] (Figure 8h). This indicates an occurrence of nucleation growth with t_d_ [56,190]. If t_d_ exceeds the optimum value, a non-uniform film with porous nature might be formed due to the dissolution of pre-adhered grains in the film [190]. This phenomenon can be experimentally observed for o-SnS, SnS_2_, and Sn_2_S_3_ films when the t_d_ varies between 2 and 10 h [177], 30–120 min [56], and 20–24 h [34], respectively. The t_d_ can considerably improve the crystallinity of the Sn_x_S_y_ films and simultaneously enhance the crystallite size. However, beyond the limit of t_d_, the crystallinity becomes poor, and the crystallite size decreases (Figure 8i) [56,146,177,190,225]. The reduction of crystallite size is due to the lowering of the van der Waals force in between crystallites because the substrate remained in the solution longer than necessary [177]. In addition to the crystallinity of films, the t_d_ also influences the phase purity of a film. At low t_d_, the released Sn^2+^ or Sn^4+^ ions are relatively low in the reaction bath compared to the available S^2−^ ions. These available S^2−^ ions are not balanced by the all released Sn^2+^ or Sn^4+^ ions, leading to the development of other secondary phases, whereas at longer t_d_ they are counterbalanced, promoting the growth of the pure phase [56].

As mentioned previously, the t_d_ directly influences the thickness of Sn_x_S_y_ films. Thus, it tremendously shows an impact on their optical transmittance/absorbance. Always, shorter t_d_ periods generate the thinnest film of high transmittance, which might be affected by abundant porosities [175,177]. Simultaneously, the more extended t_d_ periods produce thick films of high absorption [34,175,190], essential for solar cell application. The longer t_d_ period also improves the size of crystallites that can affect the optical absorption and the band gap energy of films [190].

The quantum size effect and changing barrier height (or variation in grain size) are also responsible for the variation in the band gap of films with t_d_ at other identical growth conditions [22,177]. The increase in t_d_ period reduces the band gap of o-SnS, SnS_2_, and Sn_2_S_3_ films from 1.83 eV to 1.30 eV [177], 2.95 eV to 2.80 eV [56], and 2.12 to 2.03 eV [34], respectively. In contrast, the longer t_d_ period generates greater compression impacts with the thickness in o-SnS films, which may enhance the band gap (0.82–1.22 eV) [175]. In addition to the optical gap, t_d_ shows a significant effect on the optical constants such as refractive index (n, SnS_2_:2.57–2.63, Sn_2_S_3_: 4.89–7.18) and extinction coefficient (k, SnS_2_: 0.69–0.61, Sn_2_S_3_: 0.0015–0.0019) (Figure 8j,k) [34,56]. However, no previous studies had included the variations in optical constants of o-SnS and c-SnS films with t_d_. On the other hand, the t_d_ reduces the electrical resistivity and improves the carrier density and mobility of carriers in the case of both o-SnS and SnS_2_ films [56,177] due to the improved crystallinity and suppression of secondary phases with t_d_ period. In contrast, in the case of c-SnS and Sn_2_S_3_ films, there are no reports available in the literature.

### 3.7. Other Parameters

#### 3.7.1. Substrate Nature and Its Cleaning Process Effect

Generally, thin films require proper mechanical support that provides sufficient adhesion. These supports are commonly called substrates. Substrates have a significant effect on the film properties in practice [268]. Therefore, the choice of a suitable substrate with a specific form for a thin film with a particular application is critical since the substrate must be structurally and chemically compatible with the thin film material in terms of thermal and mechanical stability [269,270]. Moreover, the substrate nature strongly affects the preferred orientation of a thin film, which plays a major role in device performance [271]. Therefore, currently, the exploration of feasible substrates has become an active research area. The CBD has the benefit of allowing thin film deposition on unevenly shaped surfaces. However, the substrate nature greatly affects the deposition process and film quality. Usually, substrates with rough surfaces have better anchoring of the initial deposit in the tiny valleys. Substrates such as glass, tin oxide (TO), indium tin oxide (ITO), and silica/quartz are relatively reactive, owing to the presence of hydroxyl surface groups. Furthermore, when the lattice of the deposited material matches well with that of the substrate, the free energy change is smaller; this facilitates fast nucleation with good morphology and structure. Although the substrate nature has more impact on the process of CBD and the deposited thin film characteristics, there are only a few studies on this area in the case of o-SnS and SnS_2_ films and no reports for c-SnS and Sn_2_S_3_ films (Table 4 and Table 5). The reports related to the effects of molybdenum (Mo), ITO, and TO and borosilicate glass substrates [204] on the properties of o-SnS films and the glass, TO, and titanium (Ti) substrates [130] on SnS_2_ films are available in the literature. At 0.01 M of Sn and S sources concentrations, both Mo and TO substrates generate o-SnS films with a better surface coverage, whereas the borosilicate glass and ITO substrates produce a discontinuous film with separate agglomerated o-SnS particles. When the concentration of sources is 0.03 M, all substrates except the borosilicate glass form a complete and uniform coverage of o-SnS films. At a high concentration of 0.09 M, all substrates produce a complete coverage of o-SnS films but with a lower adhesive nature [204]. In the case of SnS_2_, the amorphous and n-type nature films formed on the glass and Ti substrates, respectively.

In addition to the substrate nature, the cleaning process of the substrate also significantly affects the quality of thin films. Improper cleaning of substrates results in the formation of pinholes in the film, which creates major issues on the fabrication of large-area devices and produces short circuits in solar cells [272]. Unfortunately, there is a lack of research on this area for CBD deposited o-SnS, c-SnS, SnS_2_, and Sn_2_S_3_ thin films.

#### 3.7.2. Stirring Speed and Humidity Effect

In the CBD, chemical solutions with a homogeneous distribution of precursors are necessary before starting the process. Continuous mixing of the reaction solution is mandatory for realizing a uniform thin film deposition [273]. This could be achieved by stirring the solutions at appropriate speeds. At the beginning of the deposition, the stirring speed does not have a significant impact on the growth rate of thin films. However, for longer deposition times, it directly affects the growth rate. In addition, stirring with uneven speed may produce a variation in thin film uniformity and improper diffusion of complex ions toward the substrate [274], and stirring provokes precipitation and reduces the final thickness of the film. Therefore, care must be taken in stirring the solution to obtain the desired quality of thin films.

On the other hand, environmental humidity also influences the formation and physical properties of CBD processed films [273] since the CBD can be performed in an open environment where the gas–liquid interface is influenced by moisture. Even after the deposition of films, they considerably degrade because of their colloidal nature [275]. Therefore, the maintenance of environmental humidity is vital for the deposition of defect-free films. Although the control of stirring speed and environmental humidity is essential for producing quality Sn_x_S_y_ films, there is no systematic study on these effects in the literature.

### 3.8. Summary

Sn_x_S_y_ are binary metal chalcogenides that have attracted considerable attention due to their abundant, low cost, and nontoxic constituent elements. In comparison to other vacuum and chemical approaches, they may be simply synthesized utilizing a simple non-vacuum CBD methodology. Sn precursors, S precursors, and complexing agents are ideally T(II)C, TA, and TEA, respectively. The following lines are made based on the examination of published data (Table 4 and Table 5) and the explanation in Section 3.1, Section 3.2, Section 3.3, Section 3.4, Section 3.5, Section 3.6 and Section 3.7. Changes in Sn precursor concentration, complexing agent concentration, and T_b_ can be used to manipulate high-intensity plans and crystallinity. Maintaining complexing agent concentration, bath pH, and t_d_, may regulate phase transition and growth rate. Controlling S precursor and complexing agent concentrations results in good morphological, optical, and electrical characteristics. As a result, optimizing each deposition parameter is critical for producing high-quality Sn_x_S_y_ thin films for a variety of applications. However, no previous research has looked at the effect of S precursor concentration on c-SnS, SnS_2_, and Sn_2_S_3_ films; complexing agent concentration on Sn_2_S_3_ films; bath pH on the properties of SnS_2_ and Sn_2_S_3_ films; T_b_ on both SnS_2_ and Sn_2_S_3_ films; and t_d_ on c-SnS and Sn_2_S_3_ films. Furthermore, for all o-SnS, c-SnS, SnS_2_, and Sn_2_S_3_ thin films, there is a dearth of research on the substrate nature-cleaning procedure, stirring speed, and humidity influence.

According to the description in this part, it is confirmed that further research is required to improve the quality of Sn_x_S_y_ films and more studies are necessary related to the optimization of all deposition parameters. Hence, research focusing on this area is essential.

## 4. Conclusions

Sn_x_S_y_ thin films deposited with CBD are a relatively recent development, and their process–property correlations must be understood for the desired application. Further, the fabrication of single-phase o-SnS, c-SnS, SnS_2_, and Sn_2_S_3_ thin films in CBD is very condition-dependent. Additionally, it is crucial to identify and separate the o-SnS, c-SnS, SnS_2_, and Sn_2_S_3_ phases. However, until recently, there has been a dearth of detailed studies on the optimization of growth parameters. The present review outlined the background and basic properties of Sn_x_S_y_ (o-SnS, c-SnS, SnS_2_, and Sn_2_S_3_) along with the principle, nucleation, growth, and growth mechanism of Sn_x_S_y_ thin films by CBD. Furthermore, the influence of growth parameters such as precursor concentration (tin source, sulfur source, and complexing agent), bath pH, bath temperature (T_b_), deposition time (t_d_) on the phase formation, and physical properties of Sn_x_S_y_ thin films were comprehensively described. As a result, the reader should be able to prepare single-phase tin sulfide materials with ease after studying the present article. Hence, the present review should motivate readers to conduct extensive investigations on Sn_x_S_y_ films to develop cost-effective, eco-friendly, and earth-abundant tin sulfide materials to meet all future energy requirements. The connection between the physical properties of Sn_x_S_y_ thin films and their photovoltaic application will be discussed in our subsequent article.

## Figures and Tables

**Table 1:** The advantages of Sn_x_S_y_ compared to the conventional absorbers (CdTe, CIGS, and CZTS) and the buffer (CdS).

Characteristics	PV Absorbers						PV Buffers	
	CdTe	CIGS	CZTS	o-SnS	c-SnS	Sn_2_S_3_	CdS	SnS_2_
**Earth abundance**	No	No	Yes	Yes	Yes	Yes	Yes	Yes
**Eco-friendly**	No	No	Yes	Yes	Yes	Yes	No	Yes
**Band gap** **(eV)**	1.45–1.5 eV [13]	1.1–1.5 [14]	1.0–1.5 [15]	1.16–1.79 [16,17,18,19,20,21,22,23]	1.64–1.75 [24,25,26,27,28,29]	0.95–2.03 [30,31,32,33,34]	2.35–2.50 [14,35]	2.04–3.30 [36,37,38,39,40,41]
**Absorption coefficient**	>10^4^	10^5^	>10^4^	10^5^	10^5^	10^4^	–	–
**Conductivity type**	p-type	p-type	p-type	p-/n-type	p-type	p-/n-type	n-type	n-type
**Carrier density** **(cm^−3^)**	10^14^–10^17^ [35]	10^12^–10^18^ [14]	10^16^–10^18^ [42]	10^11^–10^18^ [43,44,45,46,47,48,49]	10^11^–10^18^ [29,50,51]	10^14^–10^16^ [45,52,53]	10^12^–10^18^ [14,35]	10^13^–10^17^ [54,55,56]
**Structure**	Zinc blend [13]	Chalcopyrite [14]	Kesterite [57]	Orthorhombic [58,59]	Cubic [60]	Orthorhombic [51]	Hexagonal [35]	Hexagonal [61]
**Maximum theoretical efficiency** **(%)**	~29	~29	31 [62]	31 [63]	>25 [64]	–	–	–

**Table 2:** The historical information and the applications of Sn_x_S_y_.

Tin Sulfides	Mineral Form [65,66,67]	Appearance [68]	Other Names	Discovered/Reported [69,70]	Applications [24,71,72,73,74,75,76,77,78,79,80,81,82,83,84,85,86,87,88,89,90,91]
**o-SnS**	Herzenbergite 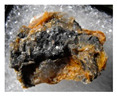	Black color with dark red–brown internal reflections.	Kolbeckine	Reported by Ramdohr from the Maria-Teresa mine (Oruro, Bolivia) in 1934.	PV, photodetectors [24], photocatalysts [71], water splitting [72], supercapacitors [83], field-effect transistors [85], sodium-ion and lithium-ion batteries [86,87], gas sensors [88], biosensors [89] thermoelectric [90], and electro chemical capacitors [91].
**SnS_2_**	Berndtite 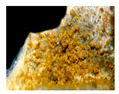	Pale yellow with intense brownish to yellow–orange internal reflections.	Mosaic gold	Discovered at the Stiepelmann mine in Arandis, Namibia, as described by Ramdohr in 1935.	PV, photocatalysts [73], water splitting [74], supercapacitors [75], field-effect transistors [76], lithium-ion and sodium-ion batteries [77,78], gas sensors [79], thin film diodes [80], and high-speed photodetectors [81].
**Sn_2_S_3_**	Ottemanite 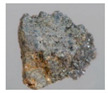	Gray with orange–brown internal reflections.	-	Reported by Moh from the Cerro de Potosi mine (Bolivia) in 1964.	PV, optoelectronic [82], thermoelectric and IR detectors [84].

**Table 3:** Optimized theoretical and experimental lattice parameters, reported optical bandgaps, and electrical parameters of Sn_x_S_y_ (Th: theoretical, Exp: experimental).

Sn_x_S_y_ Phase	Structural Properties						Optical Properties	Electrical Properties		
	Structure (Space Group)	Oxidation State of Sn	Parameters of unit cell				Optical Band Gap (eV)	Carrier Concentration (cm^−3^)	Mobility (cm^2^V^−1^s^−1^)	Resistivity (Ω-cm)
			Angles and Rule	Intercepts a (Å), b (Å), c (Å)						
				Theoretical [51]	Experimental [111,125,126]					
**o-SnS**	Orthorhombic (Pnma)	2+	α = β = γ = 90° a ≠ b ≠ c	4.251, 11.082, 3.978	4.33, 11.18, 3.98		1.16 [16], 1.30 [17], 1.32 [18], 1.35 [19], 1.42 [20], 1.43 [20], 1.48 [21], 1.70 [22], 1.79 [23].	1 × 10^11^ [43], 3.6 × 10^12^ [44], (1–1.2) × 10^15^[21,45], 1.5 × 10^16^ [46], (1–1.16) ×10^17^ [47,48], (1–3) ×10^18^ [49].	3.7 [20], 15.3 [46], 90 [43,49], 228 [44], 385 [19], 400–500 [17].	12.98 [17], 14.49 [20], 30 [16], 33.33 [18], 0.63 × 10^3^ [43], 2.1 × 10^4^ [44], (0.16–0.25) × 10^5^ [127,128].
**c-SnS**	Cubic (P2_1_3)	2+	α = β = γ = 90° a = b = c	11.506	11.603		1.64 [24], 1.66 [25], 1.67 [26], 1.73 [27], 1.74 [28], 1.75 [29].	5.87 × 10^11^ [29], 7.93 × 10^12^ [50], 6 × 10^18^ [51].	1.47 × 10^−2^ [51], 75 [50], 77.7 [29].	70 [51], 1 × 10^4^ [50], 1.37 × 10^5^ [29], 1 × 10^6^ [25], 1 × 10^7^ [28].
**SnS_2_**	Hexagonal (P$\bar{3}$ml)	4+	α = β = 90°; γ = 120˚ a = b ≠ c	3.651, 3.651, 6.015	3.638, 3.638, 5.880		2.04 [36], 2.12 [39], 2.14[129], 2.18 [37], 2.30[38], 2.35 [130], 2.40[131], 2.41 [39], 2.44[132],2.45[133] 2.50[134],2.67[135] 2.75[136], 2.80 [56], 3.08 [40], 3.30 [41].	1 × 10^13^ [54], 2 × 10^17^ [55], 6.8 × 10^17^ [56].	15 [54], 48 [56], 51.5 [55].	1.11 [55], 11.2 [56], 0.77 × 10^2^[137], 0.42 × 10^5^ [54], 0.26 × 10^7^ [138].
**Sn_2_S_3_**	Orthorhombic (Pnma)	2+ and 4+	α = β = γ = 90°	a ≠ b ≠ c	8.11, 3.76, 13.83	8.878, 3.751, 14.020	0.95 [30], 1.16 [31] 1.2 [139], 1.65 [32], 1.9 [33], 1.96 [140], 2.0 [141], 2.03 [34].	9.4 × 10^14^ [52] 1 × 10^15^ [45], 4.0 × 10^16^ [53].	20.5 [53]	0.359 [124], 7.57 [53], 0.66 × 10^2^ [45], (0.22–0.36) × 10^3^ [52,141], (0.4–2.5) × 10^5^ [137].

**Table 4:** Deposition conditions and the physical properties of ORT- and c-SnS films grown by CBD.

Sn_x_S_y_ Phase	Precursors	Complexing Gent	Deposition Parameters	Structure	Band Gap (eV)	Electrical Parameters				Ref
						Type	R (Ωcm)	µ (cm^2^V^−1^S^−1^)	N (cm^−3^)	
**o-SnS**										
o-SnS	T(II)C = 0.1 M TA = 0.1 M	TEA = 15 mL NH_3_ = 8 mL	T_b_ = 27 °C t_d_ = 20 h pH = 10.5 ± 1	Amorphous	1.51 (i)	n	–	–	–	1987 [145]
o-SnS	T(II)C = 0.025 mol SDS/AS = 0.025 mol	–	T_b_ = – t_d_ = – pH = 3,10,12	ORT(013)	1.08	p	10^7^–10^3^	–	–	1989 [36]
o-SnS	T(II)C = – TU = –	–	T_b_ = – t_d_ = – pH = –	Polycrystalline	1.3 (i)	–	–	–	–	1990 [157]
o-SnS	T(II)C = 1 g TA = 1 M	TEA = 12 mL NH_3_ = 10 mL	T_b_ = 75 °C, 25 °C t_d_ = 5 h, 40 h pH = –	Polycrystalline	1.3	p	–	–	–	1991 [128]
o-SnS	T(II)C = 2 mL TA = 1 mol L^−1^	TEA = 0.5 mL NH_3_ = –	T_b_ = 50 °C, 25 °C t_d_ = 2–4 h, 5–10 h pH = –	Crystalline	–	–	–	–	–	1991 [158]
o-SnS	T(II)C = 1 g TA = 4 mL, 8 mL	TEA = 12 mL NH_3_ = 12 mL	T_b_ = 75 °C, 25 °C t_d_ = 5 h, 40 h pH = –	–	–	–	–	–	–	1991 [159]
o-SnS	T(II)C = 1 g TA = 1 M	TEA = 12 mL NH_3_ = 10 mL	T_b_ = 60 °C t_d_ = 7 h 30 min pH = –	ORT(111)	–	–	–	–	–	1992 [160]
o-SnS	T(II)C = 1 g TA = 1 M	TEA = 12 mL NH3 = 10 mL	T_b_ = 60 °C t_d_ = 7 h 30 min pH = 9.5	ORT(111)	–	p	–	–	–	1993 [161]
o-SnS	T(II)C = 1 g TA = 1 M	TEA = 12 mL NH_3_ = 13 mL	T_b_ = 50–75 °C, t_d_ = 1.5 h, 20 h pH = –	ORT(111)	–	p	–	–	–	1994 [162]
o-SnS	T(II)C = 15 g TU = 5 g, 10 g	–	T_b_ = – t_d_ = 5 min pH = 3 S_p_ = 1.33 mm/s	ORT(040)	1.4	–	–	–	–	1999 [163]
o-SnS	T(II)C = 0.1 M SDS = 0.05 M	–	T_b_ = 80 °C t_d_ = – pH = 12	–	–	p	–	–	–	2001 [164]
o-SnS	T(II)C = 1.125 g ST = 2 M	AF	T_b_ = T_r_ t_d_ = 18 h pH = 7	ORT(111)	1.38 (d) 0.96–1.14 (i)	–	–	–	–	2003 [165]
o-SnS	T(II)C = 1 g TA = 8 mL	TEA = 12 mL NH3 = 10 mL	T_b_ = 35 °C t_d_ = 15 h pH = 9.5	ORT(111)	1.18 (d)	p	10^7^–10^4^	–	–	2003 [166]
o-SnS	T(II)C = 0.56 g SS = 0.025 M	–	T_b_ = 80 °C t_d_ = – pH = 12	ORT(111)	–	–	–	–	–	2006 [167]
o-SnS	T(II)C = 1.13 g TA = 0.1	TEA = 30 mL NH_3_ = 16 mL	T_b_ = RT 293–298 K t_d_ = 5–6 h pH = –	ORT(111)	1.17 (d) 1.12 (i)	–	10^8^–10^6^	–	–	2007 [168]
	T(II)C = 1 g TA = 1 M	TEA = 12 mL NH_3_ = 10 mL	T_b_ = 308 K t_d_ = 20 h pH = –							
o-SnS	T(II)C = 1 g TA = 1 M	TEA = 10 mL NH_3_ = 5 mL	T_b_ = 45 °C t_d_ = – pH = –	ORT(111)	1.33–1.39 (d)	–	–	–	–	2007 [169]
o-SnS	T(II)C = 1 g TA = 8 mL	TEA = 12 mL NH_3_ = 10 mL	T_b_ = 55 °C t_d_ = 8 h pH = –	ORT(111)	–	p	10^3^	90	10^11^	2008 [43]
o-SnS	T(II)C = 1.12 g ST = 0.5 M	TTA = 10 mL	T_b_ = T_r_ t_d_ = 24 h pH = 7	ORT(111)	1.1 (d)	–	10^6^	-	–	2008 [170]
o-SnS	T(II)C = 1 g TA = 8 mL	TEA = 12 mL NH_3_ = 10 mL	T_b_ = 313 K t_d_ = 8–22 h pH = –	ORT(111)	1.2–1.7 (d)	p	–	–	–	2009 [171]
o-SnS	T(II)C = 0.15 M ST = 2 M	NH_4_OH = 6 mL	T_b_ = 30 °C t_d_ = 24 h pH = 7	ORT(040)/(141)	1.31 (d)	–	–	–	–	2009 [172]
o-SnS	T(II)C = 2 × 10^−2^ M TA = 1 × 10^−2^–8 × 10^−2^ M	–	T_b_ = 80 °C t_d_ = 60 min pH = 1.87	Amorphous	–	–	–	–	–	2009 [142]
o-SnS	T(II)C = 1 g TA = 8 mL	TEA = 12 mL NH_3_ = 10 mL	T_b_ = 55 °C t_d_ = 8 h pH = –	ORT(111)	1.12 (i)	–	–	–	–	2009 [173]
o-SnS	T(II)C = 0.15 M ST = 2 M	AH = 6 mL	T_b_ = T_r_ t_d_ = 24 h pH = 7	ORT(111)	–	–	–	–	–	2009 [174]
o-SnS	T(II)C = – TA = –	TEA = – NH_3_ = –	T_b_ = 75 °C t_d_ = – pH = –	ORT(111)	0.82–1.22 (i)	–	–	–	–	2009 [175]
o-SnS	T(II)C TA = 0.1 M	TEA = 30 mL NH_4_OH = 16 mL	T_b_ = – t_d_ = 5 h pH = –	ORT(111)/(040)	1.76 (i)	–	–	–	–	2010 [176]
o-SnS	T(II)C = 1 M TA = 1 M	TEA = 10 mL TSS = 5 mL NH_3_/NH_4_Cl = 5 mL	T_b_ = 60 °C t_d_ = 2–10 h pH = 9.31	ORT(111)/(040)	1.30–1.97 (d) 0.83–1.36 (i)	p	9.9–12.3	–	–	2010 [177]
o-SnS	T(II)C = 1 M TA = 1 M	TEA = 10 mL TSS = 5 mL NH_3_/NH_4_Cl = 5 mL	T_b_ = 27 °C t_d_ = 24 h pH = 10.7	ORT(110)	1.37 (d) 1.05 (i)	p	10^5^	9 × 10^5^	–	2010 [178]
o-SnS	–	TEA = 12.5 M, 13 M	T_b_ = – t_d_ = – pH = –	–	1.93–2.16 (d)	–	–	–	–	2010 [179]
o-SnS	T(II)C = 0.95 g TA = 0.1 M	TEA = 8 mL NH_3_ = 6 mL	T_b_ = 75 °C t_d_ = 1 h pH = –	ORT(111)/(040)	1.3 (i)	p	–	–	–	2010 [180]
o-SnS	T(II)C = – TA = –	TEA, NH_3_ TTA	T_b_ = T_r_, 90 °C t_d_ = 24 h, 3 h pH = –	ORT(400)	1.1–1.9 (d)	–	–	–	–	2011 [181]
o-SnS	T(II)C = 0.2 M ST = 0.2 M	Na_2_EDTA = 25 mL of 0.2 M	T_b_ = 40–80 °C t_d_ = 30 h pH = 1.5	–	1.2–1.5 (d)	–	–	–	–	2011 [182]
o-SnS	T(II)C = 0.15 M ST = 0.15 M	Na_2_EDTA = 25 mL of 0.2 M	T_b_ = 75 °C t_d_ = 150 min pH = –	–	1.2–1.6 (d)	–	–	–	–	2011 [183]
o-SnS	T(II)C = 0.1 M ST = 0.25 M	AC = 50 mL of 0.2 M	T_b_ = 35 °C t_d_ = 10 h pH = 5, 6	ORT(111)	1.75 (d) 1.12 (i)	–	–	–	–	2011 [184]
o-SnS	T(II)C = 0.1 M ST = 0.25 M	AC = 50 mL of 0.2 M	T_b_ = 35°C t_d_ = 10 h pH = 5	ORT(111)	1.75 (d) 1.15 (i)	–	420	–	–	2011 [185]
o-SnS	T(II)C TA	TEA = – NH_3_ = –	T_b_ = 20–50 °C t_d_ = – pH = –	ORT(111)	1.15 (i) 1.35(d)	p	6.3 ± 0.1	11 ± 7	10^16^–10^17^	2011 [186]
o-SnS	T(II)C = 0.1 M ST = 0.25–0.75 M	AC = 50 mL of 0.3 M	T_b_ = 60–80 °C t_d_ = 3 h pH = 5	ORT(111)/(040))	1.01–1.26 (i)	p	10^3^	–	–	2012 [187]
o-SnS	T(II)C = – TA = –	TEA = – NH_4_Cl = –	T_b_ = 45 °C t_d_ = 5 h pH = –	ORT(111)	0.7–1.3 (i)	–	–	–	–	2012 [188]
o-SnS	T(II)C = 1 g TA = 1 M	TEA = 12 mL NH_3_ = 10 mL	T_b_ = 60 °C t_d_ = 6 h pH = 6	ORT(111)/(101)	0.9–1.1	–	10^6^–10^1^	–	–	2012 [189]
o-SnS	T(II)C = 1 M TA = 1 M	TEA = 10 mL NH_3_ = 2 mL	T_b_ = RT = 27 °C t_d_ = 24–72 h pH = 9.7	ORT(111)	1.14–1.18 (i) 1.32–1.44 (d)	–	–	–	–	2012 [190]
o-SnS	T(II)C = 0.06 M–0.12 M TA = 0.1 M	TEA = 1.85 M NH_3_ = 1.5 M	T_b_ = 30 °C t_d_ = 90 min pH = –	ORT(040)/(111)	1.5–1.95 (d)	–	–	–	–	2012 [191]
	T(II)C = 0.1 M TA = 0.1 M	TEA = 1.75–1.90 M NH_3_ = 1.5 M	T_b_ = 30 °C t_d_ = 90 min pH = –							
	T(II)C = 0.1 M TA = 0.1 M	TEA = 1.85 M NH_3_ = 1.5 M	T_b_ = 40–60 °C t_d_ = 90 min pH = –							
o-SnS	T(II)C = 1 g TA = 1 M	TEA = 12 mL NH_4_OH = 10 mL	T_b_ = 60 °C t_d_ = 6 h pH = –	ORT(111)	1.9 (d) 1.1 (i)	–	–	–	–	2013 [192]
o-SnS	T(II)C = 0.1 M TA = 0.6 M	TTA = 1 M	T_b_ = 50–70 °C t_d_ = 50 min pH = 1.5	ORT(111)	1.30–1.35 (d)	–	–	–	–	2013 [193]
o-SnS	T(II)C = 0.05–0.2 M TA = 0.4–0.7 M	Na_2_EDTA = 20 mL of 0.1 M	T_b_ = 50–80 °C t_d_ = 0.5–3 h pH = 9–12	ORT(200)	–	–	–	–	–	2013 [194]
o-SnS	T(II)C = 0.1 M ST = 0.3 M	Na_2_EDTA = 5 mL of 0.1 M TSC = 5 mL of 0.66 M	T_b_ = T_r_ t_d_ = 24 h pH = 10	–	1.50–1.90 (d)	–	–	–	–	2013 [195]
o-SnS	T(II)C = 0.5 M TU = 1 M	NH_3_ = 3 M	T_b_ = T_r_ t_d_ = 60–180 min pH = –	–	1.98–2.01(d) 1.82–1.98 (i)	p	–	–	–	2013 [196]
o-SnS	T(II)C TA = 0.1 M	TEA = 30 mL NH_4_OH = 16 mL	T_b_ = – t_d_ = 5 h pH = –	ORT(111)/(200)	1.64–1.7 (f)	–	–	–	–	2013 [197]
o-SnS	T(II)C = 0.1 M TA = 0.1 M	EDTA = 0.05 M–0.08 M NH_3_ = 1.4 M	T_b_ = – t_d_ = 3–4 h pH = –	ORT(111)/(101)	1.5–1.60 (d)	p	400	–	–	2013 [198]
o-SnS	T(II)C = 1 g TA = 1 M	TEA = 6 mL NH_3_ = 10 mL	T_b_ = – t_d_ = – pH = –	ORT(240)	1.78–1.75 (d)	–	10^9^–10^8^	–	–	2014 [199]
o-SnS	T(II)C = 1 g TA = 1 M	TEA = 12 mL NH_3_ = 10 mL	T_b_ = 20–40 °C t_d_ = 24 h pH = 11	ORT(111)	ORT 1.1 (i)	p	10^7^–10^2^	–	–	2014 [146]
o-SnS	T(II)C = 0.5 g TA = 1 M	TEA = 6 mL TSC = 0.006–0.008 M NH_3_ = 5 mL	T_b_ = 30 °C t_d_ = 24 h pH = –	ORT(111)	1.17–1.40 (d)	–	10^4^	148–228	10^12^	2014 [44]
o-SnS	T(II)C = – TA = –	TSC = –	T_b_ = 50 °C t_d_ = 2.5 h pH = 5	ORT(111)	1.25–1.83 (d) 1.1–1.65 (i)	n	10^3^	–	–	2014 [200]
o-SnS	T(II)C = 0.03 M ST = 0.03 M	TTA = 0.44 M	T_b_ = T_r_ t_d_ = 24 h pH = 7	ORT(400)	1.49–1.39 (i) 1.28–1.5 (i)	–	–	–	–	2014 [201]
o-SnS	T(II)C = 1 g TA = 1 M	TEA = 12 mL NH_3_ = 10 mL	T_b_ = 40 °C t_d_ = 17 h pH = –	ORT(111)	1.25–1.1 (i)	–	10^3^	–	–	2015 [202]
o-SnS	T(II)C = 0.1 M TA = 0.1 M	TEA = 15 mL NH_3_ = 8 mL	T_b_ = 26 °C t_d_ = 22 h pH = –	ORT(021)	1.76–3.32 (d)	–	–	–	–	2015 [203]
o-SnS	T(II)C = – ST = 0.01–0.09 M	TTA	T_b_ = 22 °C t_d_ = 24 h pH = 7	–	–	–	–	–	–	2015 [204]
o-SnS	T(II)C = 20 mL TA = 20 mL	TTA = 1 M	T_b_ = 40–80 °C t_d_ = 50 min pH = 1.5	–	1.33–1.41 (d)	–	–	–	–	2015 [17]
o-SnS	T(II)C = 0.1 M TA = 0.15 M	TSC = 0.2 M NH_3_ = –	T_b_ = 80 °C t_d_ = 4 h pH = 7	ORT(040)	1.65 (d)	p	–	–	–	2016 [205]
o-SnS	T(II)C = 1 g TA = 8 mL	TEA = 12 mL NH_3_ = 10 mL	T_b_ = 40 °C t_d_ = 10 h pH = 11	ORT(111)	ORT = 1.1 (i)	p	10^6^	–	–	2016 [25]
o-SnS	T(II)C = 0.1 M TA = 20 mL	TTA = 1 M	T_b_ = 70 °C t_d_ = – pH = –	ORT(111)	1.31–1.26 (d)	p	6–38	124	10^15^–10^16^	2016 [144]
o-SnS	T(II)C = 1 g TA = 0.3 g	TEA = 5.5 mL NH_3_ = 5 mL	T_b_ = 70 °C t_d_ = – pH = –	ORT(002)	1.14–1.75 (d)	–	–	–	–	2016 [206]
o-SnS	T(II)C = – TA = –	TTA = 1 M	T_b_ = 70 °C t_d_ = – pH = –	ORT(111)	1.3 (d)	p	38–14.2	55–23	10^15^–10^19^	2016 [123]
o-SnS	T(II)C = 0.1 M TA = 0.15 M	TSC = 0.15–0.21 M	T_b_ = 80 °C t_d_ = 4 h pH = 5.8	ORT(111)	1.64–1.1 (d)	–	–	–	–	2017 [207]
o-SnS	T(II)C = 0.1 M TA = 0.15 M	TSC = 0.2 M	T_b_ = 80 °C t_d_ = 4 h pH = 6.5–7.5	ORT(111)	1.51 (d)	–	–	–	–	2018 [27]
o-SnS	T(II)C = 1 g TA = 1 M	TEA = 312 mL NH_3_ = 10 mL	T_b_ = 40 °C t_d_ = 17 h pH = 1.5	ORT(111)	1.1 (i)	–	–	–	–	2018 [208]
o-SnS	T(II)C = 4 mmol TA = 4–8 mmol	TSC = 0.15–0.21 M	T_b_ = 80 °C t_d_ = 1–2 h pH = 0.4–1.0	ORT(111)	1.39–1.41 (d)	–	–	–	–	2018 [209]
o-SnS	T(II)C = 0.1 M TA = 0.15 M	TEA = –	T_b_ = 343 K t_d_ = 120, 240, 369 min pH = 4	ORT(013)	–	–	–	–	–	2018 [210]
o-SnS	T(II)C = 20 mL TA = 20 mL	TTA = 0.6–1.6 M	T_b_ = 70 °C t_d_ = 50 min pH = –	ORT(111)	1.28–1.45 (d)	p	38–62	29–108	1.92 × 10^15^–4.12 × 10^15^	2019 [211]
o-SnS	T(II)C = 0.1 M TA = 0.6 M	TTA = 1 M	T_b_ = 40–80 °C t_d_ = 50 min pH = 1.5	ORT(111)	1.30–1.41 (d)	p	38	55	1.5 × 10^15^–3.4 × 10^15^	2019 [212]
o-SnS	T(II)C = 1 g TA = 1 M	TEA = 18 mL NH_3_ = 10 mL	T_b_ = 40–70 °C t_d_ = 3 H pH = 10	ORT(040)	1.32–2.08 (d)	–	–	–	–	2019 [213]
o-SnS	T(II)C = 0.2 M TA = 0.4 M	TTA = 0.5 M	T_b_ = 50–80 °C t_d_ = 90 min pH = 1.5	ORT(040)	1.55–1.92 (d)	–	–	–	–	2019 [214]
o-SnS	T(II)C = 0.1 M TA = 0.15 M	TSC = 0.2 M	T_b_ = 80 °C t_d_ = 4 h pH = 5.0–6.5	ORT(111)	1.34–1.51 (d)	–	–	–	–	2019 [215]
o-SnS	T(II)C = 4 mmol TA = 6 mmol	–	T_b_ = 80 °C t_d_ = 120 min pH = 0.7	ORT(111)	1.41–1.49 (d)	–	–	–	–	2019 [216]
o-SnS	T(II)C = 2 g ST = 0.2 M	TEA = 70 mL CA = 0.4 M NH_3_ = 10 mL	T_b_ = 55 °C t_d_ = 4 h pH = 11	ORT(111)	1.33 (i)					2019 [217]
o-SnS	T(II)C = 20 mL TA = 10 mL PVA = 2 g	TTA = 0.5 M	T_b_ = 80 °C t_d_ = 45–90 min pH = 10	ORT(040)	1.55–1.79 (d)	–	–	–	–	2019 [218]
o-SnS	T(II)C = 0.1 M TA = 1 M	TEA = 10 mL TSC = 0.66 M	T_b_ = – t_d_ = – pH = 9.2–9.6	ORT(102)	1.36–1.99 (d)	–	–	–	–	2020 [219]
o-SnS	T(II)C = 0.1 mol TA = 0.4 mol	AA = 0.8 mL	T_b_ = 75 °C t_d_ = 70 min pH = 9.2–9.6	ORT(110)	–	–	–	–	–	2020 [220]
o-SnS	T(II)C = – TA = 0.1 M	TEA = – NH_3_ = 15 mL	T_b_ = 25 °C t_d_ = 4 h pH = – 200–600 °C	–	1.5–1.7 (d)	–	–	–	–	2021 [221]
o-SnS	T(II)C = 1 g TA = 0.6 g	TEA = 12 mL NH_3_ = 15 mL	T_b_ = 70 °C t_d_ = 2 h pH = 10.93	ORT(111)	1.38 (d)	–	–	–	–	2021 [222]
o-SnS	T(II)C = 4 m mol TA = 6 m mol	–	T_b_ = 80 °C t_d_ = 120 min pH = 0.7	ORT(111)	0.78–1.13 (d)	–	–	–	–	2021 [223]
o-SnS	–	–	T_b_ = 65 °C t_d_ = 3 h pH = 5.5–8.5	ORT(111)	1.41–1.75 (d)	–	–	–	–	2021 [224]
**c-SnS**										
c-SnS	T(II)C = 2.26 g TA = 10 mL	TEA = 30 mL NH_3_ = 16 mL	T_b_ = 25 °C t_d_ = 6 h pH = –	CUB (111)/(200)	1.64–1.73 (d)	p	10^5^	10^4^	10^9^	2008 [43]
c-SnS	T(II)C = 2.26 g TA = 10 mL	TEA = 30 mL NH_3_ = 16 mL	T_b_ = 25 °C t_d_ = 6 h pH = –	CUB (111)/(200)	1.7 (d)					2009 [171]
c-SnS	T(II)C = 2.26 g TA = 10 mL	TEA = 30 mL NH_3_ = 16 mL	T_b_ = 25 °C t_d_ = 6 h pH = –	CUB (111)/(200)	1.7 (d)	–	–	–	–	2009 [173]
c-SnS	T(II)C = – TA = 0.1 M	TEA = 8964 g NH_4_OH = 15 M	T_b_ = 25 °C t_d_ = 2–4 h 30 min pH = –	CUB (111)/(200)	1. 7 (d)	–	–	–	–	2011 [225]
c-SnS	T(II)C = – TA = 0.1 M	TEA = – NH_4_OH = 15 M	T_b_ = 25 °C t_d_ = – pH = –	–	–	–	–	–	–	2011 [226]
c-SnS	T(II)C = – TA = 0.1 M	TEA = 8964 g NH_4_OH = 15 M	T_b_ = 25 °C t_d_ = – pH = –	CUB (111)/(200)	1.76 (d) 1.44–1.51 (d)	–	–	–	–	2012 [227]
c-SnS	T(II)C = – TA = 0.1 M	TEA = 8964 g NH_4_OH = 15 M	T_b_ = 25 °C t_d_ = – pH = –	CUB (111)/(200)	1.76 (d)	–	–	–	–	2012 [228]
c-SnS	T(II)C = 1 g TA = 1 M	TEA = 12 mL NH_3_ = 10 mL	T_b_ = 20–40 °C t_d_ = 24 h pH = 11	CUB (111)/(200)	1.67 (d)	p	10^7^–10^2^	–	–	2014 [146]
c-SnS	T(II)C = 2.26 g TA = 0.1 M	TEA = 30 mL NH_3_ = 16 mL	T_b_ = 17 °C t_d_ = 10 h pH = –	CUB (222)/(400)	1.74 (d)	–	–	–	–	2015 [28]
c-SnS	T(II)C TA = 0.1 M	TEA = 0.1 M NH_4_OH = 15 M	T_b_ = 25 °C t_d_ = – pH = –	CUB(111)/(200)	1.70 (d)	–	–	–	–	2015 [229]
c-SnS	T(II)C = 2.26 g T(II)C = 0.1 M	TEA = 30 mL NH_3_ = 16 mL	T_b_ = 17 °C t_d_ = 15 h pH = 11	CUB(222)/(400)	1.73 (d)	–	10^3^	–	–	2016 [230]
c-SnS	T(II)C = 2.26 g TA = 10 mL	TEA = 30 mL NH_3_ = 16 mL	T_b_ = 17 °C, 10 °C t_d_ = 4 h, 18 h pH = 11	CUB(222)/(400)	1.66–1.72 (d)	p	10^6^	–	–	2016 [25]
c-SnS	T(II)C = 0.1 M TA = 0.1 M	TEA = 30 mL NH_3_ = 16 mL	T_b_ = 25 °C t_d_ = 6 h pH = 11	CUB(222)/(400)	–	–	–	–	–	2016 [231]
c-SnS	T(II)C = 2.26 g TA = 0.1 M	TEA = 30 mL NH_3_ = 16 mL	T_b_ = 17 °C t_d_ = 10 h pH = 11	CUB(222)/(400)	–	–	–	–	–	2016 [232]
c-SnS	T(II)C = 2.26 g ST = 1 M	EDTA = 20 mL of 0.5 M	T_b_ = 25–65 °C t_d_ = 6 h pH = 10.5	CUB(222)/(400)	1.74–1.68 (d)	p	10^5^–10^4^	8.98–28.6	10^12^–10^13^	2016 [50]
c-SnS	T(II)C = 2.26 g ST = 1 M	EDTA = 15–25 mL of 0.5 M NH_3_ = 5 mL	T_b_ = 45 °C t_d_ = 6 h pH = 10.5 S_p_ = –	CUB(222)/(400)	1.67–1.73 (d)	p	10^5^–10^4^	0.34–28.6	10^14^–10^12^	2016 [26]
c-SnS	T(II)C = 0.1 M TA = 0.15 M	TSC = 0.2 M	T_b_ = 80 °C t_d_ = 4 h pH = 7	CUB(222)/(400)	1.64 (d)	–	–	–	–	2017 [24]
c-SnS	T(II)C = 0.1 M TA = 0.1 M	TEA = 30 mL NH_3_ = 16 mL	T_b_ = 17 °C, 80 °C t_d_ = 3 h, 21 h pH = –	–	–	–	–	–	–	2017 [233]
c-SnS	T(II)C = 0.1 M ST = 0.125 M	EDTA = 0.1 M	T_b_ = 45 °C t_d_ = 6 h pH = –	CUB(222)/(400)	1.67–1.75 (d)	p	10^5^–10^4^	5.22–77.7	10^11^–10^13^	2017 [29]
c-SnS	T(II)C = 0.1 M TA = 0.15 M	TSC = 0.2 M	T_b_ = 80 °C t_d_ = 4 h pH = 6.5–7.5	CUB(222)/(400)	1.64–1.73 (d)	–	–	–	–	2018 [27]
c-SnS	T(II)C = 0.1 M TA = 0.15 M	TSC = 0.2 M	T_b_ = 80 °C t_d_ = 4 h pH = 7	CUB(222)/(400)	1.5 (d)	–	–	–	–	2018 [234]
c-SnS	T(II)C = 0.5 M TA = 0.5 M	TEA = 30 mL NH_3_ = 16 mL	T_b_ = 35 °C t_d_ = 4 h pH = 9.78	CUB(222)/(400)	1.74 (d)	–	–	–	–	2018 [235]
c-SnS	T(II)C = 2.26 g TA = 0.1 M	TEA = 30 mL NH_3_ = 16 mL	T_b_ = 17 °C, 80 °C t_d_ = 3 h, 21 h pH = –	CUB(222)/(400)	1.76 (d)	–	–	–	–	2018 [236]
c-SnS	T(II)C = 2.26 g TA = 10 mL	TEA = 30 mL NH_3_ = 16 mL	T_b_ = 17–8 °C t_d_ = 3–21 h pH = –	CUB(222)/(400)						2019 [217]
c-SnS	T(II)C = 0.04 M TA = 0.08 M	TEA = 1.1 M NH_3_ = 9.5 mL	T_b_ = 30 °C t_d_ = 4 h pH = –	CUB(222)/(400)	1.74 (d)	–	–	–	–	2020 [237]
c-SnS	T(II)C = 0.1 M TA = 0.15 M	TSC = 0.2 M	T_b_ = – t_d_ = - pH = –	CUB(222)/(400)	–	–	–	–	–	2020 [238]
c-SnS	T(II)C = 1 g TA = 0.3 g	TEA = 5.5 mL NH_3_ = 5 mL	T_b_ = 24 °C t_d_ = 4.25 h pH = 9.25	CUB(222)/(400)	1.70–1.74 (d)	–	10^3^–10^4^	–	–	2020 [239]
c-SnS	T(II)C = 0.2 M TA = 0.1 M	TEA = 5.5 mL NH_3_ = 5 mL	T_b_ = 17–8 °C t_d_ = 3–21 h pH = 11	CUB(222)/(400)	1.76 (d)	p	10^8^	–	–	2020 [240]
c-SnS	T(II)C = 2.25 g ST = 0.1 M	EDTA = 0.5 M NH_3_ = 5–7.5 mL	T_b_ = 50 °C t_d_ = 6 h pH = 10.3	CUB(222)/(400)	1.75–1.8 (d)	p	10^3^–10^4^	15–75	10^12^–10^13^	2020 [241]
c-SnS	T(II)C = 1 g TA = 0.6 g	TEA = 12 mL NH_3_ = 15 mL	T_b_ = 70 °C t_d_ = 2 h pH = 8.24	CUB(200)	1.72 (d)	–	–	–	–	2021 [222]
c-SnS	T(II)C = 1.21 g TA = 0.5 M	TTA = 1 M	T_b_ = 80 °C t_d_ = 2–6 h pH = 5–8	CUB(222)/(400)	1.72–1.90 (d)	–	10^7^–10^8^	–	–	2021 [242]
c-SnS	T(II)C = 0.5 g TA = 1 M	NTA = 0.6 M	T_b_ = 40 °C t_d_ = 90–182 min pH = 10	CUB(222)/(400)	1.77–1.81 (d)	–	10^6^	–	–	2021 [243]
c-SnS	T(II)C = 0.01 mol TA = 0.1 M	TEA = 0.6 M	T_b_ = 17–8 °C t_d_ = 3–21 h pH = 10	CUB(222)/(400)	1.70–1.80 (d)	–	–	–	–	2021 [244]

**Table 5:** Deposition conditions and the physical properties of SnS_2_ and Sn_2_S3 films grown by CBD.

Sn_x_S_y_ Phase	Precursors	Complexing Agent	Deposition Parameters	Structure	Band Gap (eV)	Electrical Parameters				Ref
						Type	R (Ωcm)	µ (cm^2^V^−1^S^−1^)	N (cm^−3^)	
**SnS_2_**										
SnS_2_	T(II)C = 0.025 mol SDS/AS = 0.025 mol	–	T_b_ = – t_d_ = – pH = 3, 10, 12	–	2.04	SnS_2_-n	10^7^–10^3^	–	–	1989 [36]
SnS_2_	Tin-ingots (99.9%) ST = 10 mL	–	T_b_ = T_r_ t_d_ = 2 h pH = –	Amorphous	2.35 (d)	n	10^3^–10^4^	–	–	1990 [130]
SnS_2_	Tin ingots (99.9%) ST = 10 mL	–	T_b_ = 27 °C t_d_ = – pH = 1.4	Amorphous	2.20 (i)	n	10^7^–10^8^	–	–	1992 [147]
SnS_2_	T(II)C = 1.13 g TA = 0.1 M	EDTA = 25 mL NH_3_ = 15 mL	T_b_ = T_r_ t_d_ = 10–120 min pH = 10	–	2.3 (d)	n	4 × 10^−1^	–	–	1997 [38]
SnS_2_	T(II)C = 15 g TU = 5 g, 10 g	–	T_b_ = – t_d_ = 5 min pH = 3 S_p_ = 1.33 mm/s	HEX(001)	2.05 (i)	–	–	–	–	1999 [163]
SnS_2_	TC(IV) = 0.02 mol TA = 0.5 mol L^−1^	CA = 0.375, 0.5, 0.625 mol/L	T_b_ = 35 °C t_d_ = – pH = 1.3	–	2.40 (d)	–	–	–	–	2011 [131]
SnS_2_	T(II)C = 1 g TA = 0.5 M	TEA = 24 mL NH_3_ = 12 mL–20 mL	T_b_ = 60 °C t_d_ = 2 h pH = –	HEX(001)	3.3–3.7 (d)	–	–	–	–	2012 [41]
SnS_2_	T(II)C = 0.8 M TA = 0.5 M	TEA = 3.75 M NH_3_ = 12 mL	T_b_ = 60 °C t_d_ = – pH = –	HEX(001)	2.8–3.0 (d)	–	–	–	–	2013 [245]
SnS_2_	T(II)C = 2.26 g ST = 1 M	EDTA = 20 mL of 0.5 M NH_3_ = 5 mL	T_b_ = 45 °C t_d_ = 6 h pH = –	HEX(001)	2.58 (d)	–	–	–	–	2017 [246]
SnS_2_	T(II)C TA	TTA = 1 M	T_b_ = – t_d_ = 30–120 min pH = –	HEX(001)	2.95–2.80 (d)	n	11.2	48	10^17^	2017 [56]
SnS_2_	T(II)C = 0.84 g TA = 0.5 M	TEA = 24 mL NH_3_ = 16 mL	T_b_ = 60 °C t_d_ = 2 h pH = –	–	–	–	–	–	–	2018 [247]
SnS_2_	T(II)C = 0.1 M TA = 0.1 M	TTA = 0.1 M	T_b_ = 60 °C t_d_ = 6 h pH = –	HEX(001)	2.25–2.53 (d)	–	–	–	–	2019 [248]
**Sn_2_S_3_**										
Sn_2_S_3_	T(II)C = 1 M TA = 1 M	TEA = 10 mL	T_b_ = 30 °C t_d_ = 20–24 h pH = 10.7	ORT(131)	2.03–2.12 (d)	–	–	–	–	2012 [34]
Sn_2_S_3_	T(II)C = 1.4 g TA = 1 M	TEA = 30 mL NH_3_ = 50 mL	T_b_ = RT t_d_ = 24 h pH = –	ORT(211)	1.2 (d)	–	–	–	–	2012 [139]
Sn_2_S_3_	T(II)C = 0.05 M SDS = 0.05 M	–	T_b_ = – t_d_ = – pH = –	ORT(021)	1.3 (d)	–	–	–	–	2018 [249]
Sn_2_S_3_	T(II)C = 0.1 M TA = 0.1 M	TEA = 30 mL NH_3_ = 16 mL	T_b_ = 17 °C t_d_ = 15 h 450 °C (S-powder: 15 mg), 5–75 min	ORT(211)	1.75 (d)	p	10^4^	6 × 10^−6^	–	2020 [250]

## Data Availability

Not applicable.

## Abbreviations

**Abbreviation**	**Chemical Name**
A	Ammonia
AA	Acetic acid
AC	Ammonium citrate
ACE	Acetone
AF	Ammonium fluoride
AH	Ammonium hydroxide
ALD	Atomic layer deposition
AS	Ammonium sulfide
BT	Baking temperature
CA	Citric acid
CALPHAD	CALculation of PHAse diagram
CBD	Chemical bath deposition
CBM	Conduction band minimum
CBO	Conduction band offset
CSS	Close space sublimation
CUB	Cubic
DDT	Dodecanethiol
DIW	Deionized water
DW	Distilled water
EDS	Energy-dispersive X-ray spectroscopy
EDTA	Ethylenediaminetetraacetic acid
EL	Electrolyte
FF	Fill factor
G	Glass
GA	Glacial acetic acid
GIXRD	Grazing incidence X-ray diffraction
HCL	Hydrochloric acid
HEX	Hexagonal
HH	Hydrazine hydrate
HWVD	Hot wall vapor deposition
ITO	Indium tin oxide
JCPDS	Joint committee on powder diffraction standards
Li	Lithium
MeOH	Methanol
Mo	Molybdenum
Na	Sodium
Na_2_EDTA	Disodium ethylenediaminetetraacetate
NTA	Nitriloacetic acid
ODE	Ooctadecene
OLA	Oleylamine
ORT	Orthorhombic
PG	Propylene glycol
PL	Photoluminescence
QE	Quantum efficiency
RS	Rock salt
SAED	Selected area electron diffraction
SCR	Space charge region
SDS	Sodium sulfide
Si	Silicon
SILAR	Successive ionic layer adsorption and reaction
SIMS	Secondary ion mass spectrometry
SS	Stainless steel
SnS	Tin monosulfide
SnS_2_	Tin disulfide
Sn_2_S_3_	Tin sesquisulfide
ST	Sodium thiosulfate
TA	Thioacetamide
T(II)C	Tin (II) chloride dehydrate
TC(IV)	Tin(IV) chloride pentahydrate
TEA	Triethanolamine
TEM	Transmission electron microscopy
Ti	Titanium
TO	Tin oxide
TOP	Trioctylphosphine oxide
T_r_	Room temperature
TSC	Trisodium citrate
TTA	Tartaric acid
TU	Thiourea
UAED	Ultrasound-assisted electrodeposition
VBM	Valence band maximum
XRD	X-ray diffraction
ZB	Zinc blended
